# Orbivirus NS4 Proteins Play Multiple Roles to Dampen Cellular Responses

**DOI:** 10.3390/v15091908

**Published:** 2023-09-12

**Authors:** Fauziah Mohd Jaafar, Mourad Belhouchet, Baptiste Monsion, Lesley Bell-Sakyi, Peter P. C. Mertens, Houssam Attoui

**Affiliations:** 1 UMR1161 VIROLOGIE, INRAE, Ecole Nationale Vétérinaire d’Alfort, ANSES, Université Paris-Est, 94700 Maisons-Alfort, France; baptiste.monsion@vet-alfort.fr; 2 Division of Structural Biology, Henry Wellcome Building for Genomic Medicine, Oxford OX3 7BN, UK; mourad.belhouchet@outlook.com; 3 Department of Infection Biology and Microbiomes, Institute of Infection, Veterinary and Ecological Sciences, University of Liverpool, 146 Brownlow Hill, Liverpool L3 5RF, UK; lsakyi@liverpool.ac.uk; 4 One Virology, The Wolfson Centre for Global Virus Research, School of Veterinary Medicine and Science, University of Nottingham, Sutton Bonington Campus, Loughborough, Leicestershire LE12 5RD, UK; peter.mertens@nottingham.ac.uk

**Keywords:** orbivirus, bluetongue virus, Great Island virus, St. Croix River virus, NS4, innate responses

## Abstract

Non-structural protein 4 (NS4) of insect-borne and tick-borne orbiviruses is encoded by genome segment 9, from a secondary open reading frame. Though a protein dispensable for bluetongue virus (BTV) replication, it has been shown to counter the interferon response in cells infected with BTV or African horse sickness virus. We further explored the functional role(s) of NS4 proteins of BTV and the tick-borne Great Island virus (GIV). We show that NS4 of BTV or GIV helps an E3L deletion mutant of vaccinia virus to replicate efficiently in interferon-treated cells, further confirming the role of NS4 as an interferon antagonist. Our results indicate that ectopically expressed NS4 of BTV localised with caspase 3 within the nucleus and was found in a protein complex with active caspase 3 in a pull-down assay. Previous studies have shown that pro-apoptotic caspases (including caspase 3) suppress type I interferon response by cleaving mediators involved in interferon signalling. Our data suggest that orbivirus NS4 plays a role in modulating the apoptotic process and/or regulating the interferon response in mammalian cells, thus acting as a virulence factor in pathogenesis.

## 1. Introduction

Twenty-two virus species have been officially recognised by the International Committee on Taxonomy of Viruses (ICTV) within the genus *Orbivirus,* one of six genera classified within the family *Sedoreoviridae* (order *Reovirales*), although additional orbivirus isolates/strains may represent several additional species [1,2]. The orbiviruses are arthropod-borne and can be transmitted between their vertebrate hosts by the bite of ‘vector-competent’ hematophagous arthropods, in which they also replicate. These vectors include *Culicoides* midges, ticks, phlebotomine flies and anopheline and culicine mosquitoes (depending on the virus species) [3,4]. It is also possible for orbiviruses to be transmitted vertically in their vertebrate hosts and in some cases transmitted horizontally by direct contact [5,6]. Collectively the orbiviruses have a wide host range that includes ruminants, equids, humans and other mammals, as well as marsupials, reptiles and birds [3,4,7]. The only known exception is *St. Croix River virus* (SCRV), which persistently infects some ixodid tick cell lines but has no known vertebrate host [8,9,10]. The most economically important orbiviruses, which infect livestock species, include *Bluetongue virus* (BTV) (the *Orbivirus* ‘type-species’); *African horse sickness virus* (AHSV) and *Epizootic hemorrhagic disease virus* (EHDV), all of which are transmitted by *Culicoides* biting midges [11].

The orbivirus genome consists of 10 segments of linear double-stranded RNA (dsRNA), which are identified as Seg-1 to Seg-10 in order of decreasing size. The coding assignments for the genome segments of BTV were previously determined; identifying seven structural proteins VP1-VP7 and five non-structural proteins NS1-NS5 [11,12,13]. NS4 and NS5 were the latest proteins to be identified. Their existence was initially suspected using bioinformatic analyses and were further detected in orbivirus-infected cells [11,13,14,15,16]. NS4 exhibits significant sequence and size diversity between members of the different *Orbivirus* species [11,17] and is believed to represent a virulence determinant countering the interferon type I response [15]. NS5 is a nucleic acid binding protein that helps to suppress host-cell protein synthesis, while maintaining ribosome function, enhancing expression of viral proteins and virus replication [13].

In this study, we report further functional characterisation of the roles played by the NS4 proteins of orbiviruses. Our results show that NS4 of BTV or Great Island virus (GIV) complements the lack of the E3L gene product, an antagonist of interferon/PKR, in mammalian cells infected with an E3L-defective vaccinia virus. E3L is also known as a suppressor of RNA silencing [18,19]. Interferons are known to induce programmed cell death (apoptosis) in tumoral cells [20,21,22,23] and pro-apoptotic caspases have been previously shown to suppress the type I interferon response by cleaving cGAS, MAVS, and IRF3 [24,25,26]. Our results show that ectopically expressed NS4 of BTV interacts with active caspase 3 in mammalian cells and by doing so, it likely contributes to dampening the interferon type I response. 

## 2. Materials and Methods

### 2.1. Ethics Statement 

Animal experimentation protocols were approved by the Ethics Committee for animal experimentation of Anses-EnvA-UPEC (project licence Number: 19-028). 

### 2.2. Cell Lines and Viruses

Baby hamster kidney BSR cells (a clone of BHK-21 cells, [27]), human uterine adenocarcinoma HeLa cells (ATCC CRM-CCL-2), human embryonic kidney HEK293 cells (ATCC CRL-1573) and mouse fibroblast L929 cells (ATCC CCL-1) were grown at 37 °C in Dulbecco’s modified Eagle’s medium (DMEM), supplemented with 10% foetal bovine serum (FBS) and 100 IU of penicillin/100 µg of streptomycin (pen/strep) per mL, under 5% CO_2_. KC cells, derived from *Culicoides sonorensis* [28], were grown in ambient air at 28 °C in Schneider’s Drosophila medium supplemented with 10% FBS and pen/strep. *Ixodes scapularis* IDE2 cells [29], persistently infected with SCRV [8], were grown in ambient air at 28 °C in L-15B medium supplemented as described previously [30]. Chicken embryo fibroblasts (CEF) were prepared as previously described [31]. 

BTV-1RG_C7_ is an attenuated strain of BTV serotype 1, previously derived by reverse genetics based on the genome sequence of the BTV-1 reference strain [32], although the basis for its attenuation has not yet been determined. An E3L-defective vaccinia virus (VV) Copenhagen strain (VVC) designated VP1080, whereby E3L was replaced by a β-galactosidase [33], was kindly provided by Prof. Bertram Jacobs, Arizona State University. Great Island virus (GIV) [14] was kindly provided by Prof. Robert Tesh, University of Texas, Galveston. Encephalomyocarditis virus (EMCV) was previously described [34]. Mammalian orthoreovirus 3 (MRV3, ATCC VR-824) was obtained from the American type cell culture collection (ATCC, Manassas, VA, USA).

A deletion mutant of the NS4 open reading frame was previously generated using the BTV-1RG_C7_ genetic-backbone and was designated BTV-1∆NS4 [13]. All eight in-frame ATG codons in the NS4 ORF (positions 182-184, 242–244, 248–250, 323–325 and 338–340 of Seg-9) were mutated to ACG. None of these changes altered the amino acid sequence of VP6 encoded by the overlapping ORF of Seg-9. 

### 2.3. Cloning ORFs Encoding Viral Proteins

The ORFs of several viral proteins were PCR-amplified using primers (described in Table S1) containing EcoRI and NotI sites to facilitate cloning into plasmids pGEX-4T-2 for bacterial expression or pCI-neo for mammalian expression. These ORFs include BTV-1 NS4 (accession number FJ969727), GIV NS4 (accession number HM543473), mammalian orthoreovirus 3 (MRV3) sigma3 protein (accession number HM159622), P19 protein of the *Tombusvirus* carnation Italian ringspot virus (CIRV) (accession number X85215) and BTV-1 VP3 (accession number DQ186792). The ORF of SCRV NS4 in genome segment 9 (accession number AF145406) is interrupted by an in-frame TGA stop codon at position 215–217 [11]. The stop codon was mutated to AGA encoding an arginine, thus restoring the full-length ORF/viral protein (Figure S1). The choice of the mutation was based on the multiple sequence alignment of insect-borne and tick-borne orbiviruses, which indicates that this position is likely an arginine.

The bacterial expression plasmid pGEX-4T-2 was modified to replace the thrombin cleavage site with a 3C protease cleavage site. Three PCR amplicons of ORFs (generated using primers shown in Table S1) were cloned into pGEXT-4T-2 to express the proteins GST-TAT-NS4BTV1-6xHis, GST-TAT-HA-VP3BTV1-6xHis or GST-TAT-HA-NS4SCRV-6xHis. The expressed, N-terminal GST fused proteins were purified by glutathione affinity chromatography. The 3C cleavage site, located between GST and the target proteins, allows the release of the N-terminal TAT-tagged and C-terminal 6XHis tagged proteins. 

The viral proteins, including both native non-tagged NS4 and C-terminal 6xHis tagged NS4 of BTV-1 and GIV, sigma3 of MRV, or P19 of CIRV were expressed in mammalian cells.

Primers were used to PCR-amplify the corresponding ORFs, and PCR products were double-digested with restriction enzymes (as indicated in Table S1) and gel-purified using a Geneclean kit (MP Biomedicals, Illkirch, France). Plasmids and PCR products were ligated overnight (O/N) at 16 °C using T4 DNA ligase (Roche, Basel, Switzerland) to generate the bacterial expression plasmids pGEX-TAT-NS4-6xHis, pGEX-TAT-HA-VP3BTV1-6xHis and pGEX-TAT-HA-NS4SCRV-6xHis and the mammalian expression plasmids pCIBTV1NS4, pCIGIVNS4, pCIBTV1NS4-6xHis, pCIP19-6xHis, pCIsigma3 and pCIBTV1NS2. Recombinant plasmids were used to transform XL1-Blue bacteria (Agilent Technologies, Les Ulis, France). Clones were recovered and grown in LB broth containing ampicillin. The plasmids were subsequently purified using a Qiaquick plasmid miniprep kit (Qiagen, Les Ulis, France) and sequenced.

### 2.4. Bacterial Expression and Purification of Recombinant Proteins

Plasmids pGEX-TAT-NS4BTV-6xHis, pGEX-TAT-HA-VP3BTV1-6xHis or pGEX-TAT-HA-NS4SCRV-6xHis were used to transform BL21(DE3) bacteria. Recombinant clones were used for bacterial protein expression of GST-TAT-NS4BTV1-6xHis, GST-TAT-HA-VP3BTV1-6xHis and GST-TAT-HA-NS4SCRV-6xHis for 8 h at 37 °C. Insoluble fractions of TAT-NS4BTV1-6xHis, TAT-HA-VP3BTV1-6xHis or TAT-HA-NS4SCRV-6xHis proteins were solubilised as previously described [35]. Briefly, the inclusion bodies were solubilised in 50 mM CAPS (3-[cyclohexylamino]-1-propane-sulfonic acid), 1 mM dithiothreitol, and 0.3% Sarkosyl and dialysed O/N against 20 mM Tris-HCl (pH 8.5). The fusion proteins were cut with 3C protease at 16 °C O/N to cleave off the glutathione *S*-transferase (GST) moiety. The resulting TAT-NS4BTV1-6xHis, TAT-HA-VP3BTV1-6xHis and TAT-HA-NS4SCRV-6xHis proteins were further purified and used to transduce cells as previously described [36,37].

### 2.5. Western Blot

Protein analyses by 10% SDS-PAGE and electroblotting onto nitrocellulose membranes were performed as previously described [11,36,38]. The primary antibodies used for Western blotting are listed in Table S2. Anti-mouse (Beckman Coulter, Villepinte, France) and anti-rabbit (Sigma, St. Louis, MO, USA) peroxidase-conjugated secondary antibodies were diluted at 1/500 in 5% skimmed milk powder prepared in TBS (TBS: 25 mM Tris/HCl, 150 mM NaCl, 2 mM KCl, pH 7.4) containing 0.1% Tween-20 (TBST). Streptavidin peroxidase (PI21130, Thermo Fisher Scientific, Waltham, MA, USA), for the detection of biotin-labelled proteins, was diluted 1/1000 in TBST.

### 2.6. Interferon Beta (IFNβ) Luciferase Reporter Assay

The effect of NS4 on IFNβ was assessed using a previously described IFNβ luciferase reporter assay [39]. An IFNβ promoter was PCR-amplified from DNA extracts of HEK293 cells using primers IFNβ-PromKpnIfor/IFNβ-PromXhoIrev (Table S1) (designed from the IFNβ gene elements: accession number EF064725). The PCR product was cloned into the pGL3 vector (Promega, Madison, WI, USA) after their co-digestion with both KpnI and XhoI restriction enzymes. The resulting plasmid was designated pGL3-IFNβ-FFLuc. For normalisation of the luciferase assay, we used the pRL-SV40 plasmid encoding *Renilla* luciferase under the control of the SV40 promoter (Promega, Madison, WI, USA). Plasmids were transfected into HEK293 cells in 24-well plates using Lipofectamine 2000, as described by the manufacturer.

To induce the interferon response, HEK293 cells were transfected with plasmid pCAGGS-Flag-2CARD-RIG-I (a gift from Prof A. Garcia-Sastre, Icahn School of Medicine at Mount Sinai, New York, NY, USA). Positive control experiments were conducted by simultaneously transfecting cells with 150 ng of pCAGGS-Flag-2CARD-RIG-I, 150 ng of pGL3-IFNβ-Luc and 150 ng of empty pCI-neo. Negative controls were performed using 150 ng of empty pCAGGS, 150 ng of pGL3-IFNβ-FFLuc and 150 ng of empty pCI-neo. 

The effects of NS4 were tested by simultaneously transfecting 150 ng of pCAGGS-Flag-2CARD-RIG-I, 150 ng of pGL3-IFNβ-FFLuc and 150 ng of pCIBTV1NS4 or pCIGIVNS4. Sigma3, a known interferon antagonist, was included as a control by transfecting cells with 150 ng of pCAGGS-Flag-2CARD-RIG-I, 150 ng of pGL3-IFNβ-FFLuc and 150 ng of pCI-sigma3. The assays were run in triplicate and all cells were also co-transfected with 150 ng of pRL-SV40 plasmid which served for normalisation. At 16 h post-transfection, cells were harvested by scraping, pelleted by centrifugation at 1000× *g* for 5 min at 4 °C and the supernatant discarded. Pellets were dissolved in 200 µL of M-PER (mammalian protein extraction reagent, Thermo Fisher Scientific, Waltham, MA, USA) by gentle shaking for 30 min. The lysate was centrifuged at 10,000× *g* for 5 min and supernatants were used to determine luciferase readings using the Dual-Luciferase^®^ Reporter Assay System (Promega, Madison, WI, USA), as directed by the manufacturer. Data are shown as the mean normalised luciferase activity of triplicates ± S.E. 

### 2.7. Gene Expression Assays (Real-Time PCR)

Total RNA was extracted from GIV-infected BSR cells, using TRIzol (Thermo Fisher Scientific, Waltham, MA, USA), and the ssRNA precipitated by 2M LiCl, as previously described [40]. The dsRNA was further purified by treating with 0.5 µg/mL of RNAse A (Roche, Basel, Switzerland) in 2XSSC (300 mM NaCl and 30 mM sodium citrate, pH 7.4) [41,42] at 37 °C for 30 min, to remove remaining traces of ssRNA. The RNAse A was subsequently removed by shaking with phenol–chloroform–isoamyl alcohol (25:24:1, Sigma, St. Louis, MI, USA) and the dsRNA was precipitated using 2.5 volumes of isopropanol and 0.5 volumes of ammonium acetate 7.5 M [40]. The resulting dsRNA pellet was dissolved in RNase-free water and analysed by PAGE. 

HeLa cells were grown in 12-well plates. Experiments were conducted in triplicate wells and repeated at three separate occasions. Six groups of HeLa cells were included in each experiment. 

One group was used as a negative control (mock transfected). Another group was used as a positive control and was transfected with dsRNA (1 µg/well) using Lipofectamine 2000 (Thermo Fisher Scientific, Waltham, MA, USA), as directed by the manufacturer. A third group was transfected twice (0 h and 6 h) with the plasmid pCIGIVNS4 or pCIBTV1NS4. A fourth group was transfected twice (0 h and 6 h) with the plasmid pCIGIVNS4 or pCIBTV1NS4, 24 h prior to dsRNA transfection. A fifth group was transfected twice (0 h and 6 h) with the plasmid pCIBTV1NS2. A sixth group (used as a control of specificity) was transfected twice (0 h and 6 h) with the plasmid pCIBTV1NS2 24 h prior to dsRNA transfection. At 8 h following the second transfection, cells were washed three times with 1 mL of ice-cold PBS, harvested by scraping, pelleted by centrifugation at 1000× *g* for 5 min at 4 °C, and the supernatant was discarded. Total RNA was extracted from the pellets using TRIzol. The extracted RNA was converted into cDNA in the presence of random hexanucleotide primers as previously described [40,43]. The relative levels of expression were assessed using proprietary real-time PCR assays (Table 1) to determine the amounts of mRNA derived from different genes, as described by the manufacturer (Thermo Fisher Scientific, Waltham, MA, USA). 

To evaluate the effect of NS4 on Dicer-2 mRNA expression levels, KC cells were transduced with TAT-NS4-6xHis of BTV-1. Primers CulicoDcr-2For and CulicoDcr-2Rev (Table S3) (which amplify a 206 bp long amplicon) were designed from a transcript of *C. sonorensis* (accession GAWM01016560.1), which we identified as encoding Dicer-2 (Dcr-2). These were used for real-time PCR assays, together with a QuantiTect SYBR Green PCR Kit (Qiagen, Les Ulis, France) to assess levels of Dicer-2 mRNA synthesis.

Primers Act1CulicoFor and Act1CulicoRev (Table S3) were designed to amplify a 164 bp-long amplicon from the sequence of the Actin-1 transcript of *C. sonorensis* (accession number AF443615). These were used as a control in RT-PCR assays, to assess the effect of NS4 on overall host transcription. All results were normalised to 18S rRNA and expressed as fold change (calculated by the ΔΔCt method) in gene expression compared to the non-transduced control. 

Levels of NS4 mRNA were assessed using real-time PCR primers and probes for the BTV NS4 ORF (NS4BTfor/NS4BTrev/NS4BTProb) and GIV NS4 (NS4GIVfor/NS4GIVrev/NS4GIVProb) (Table S3), as previously described [34].

### 2.8. Replication of Vaccinia-∆E3L and Vaccinia-∆E3L/NS4 Recombinant Viruses in Interferon-Treated Cells

The VV E3L-deletion mutant VP1080 was used to construct recombinant VV expressing NS4 proteins of BTV-1 or GIV, in order to assess whether they could complement E3L deletion. The ORFs encoding these two proteins were cloned into a synthetic shuttle plasmid (ShuttleVacc: previously described [13]) used for inserting transgenes at the E3L locus of VV by homologous recombination, thereby inserting the transgene of interest at the E3L locus. ShuttleVacc also contains a yellow fluorescent protein (YFP) under the control of the EMCV IRES. YFP facilitates the identification of recombinant VV clones. ORFs of NS4 of BTV-1 or GIV were cleaved from pCIBTV1NS4 and pCIGIVNS4 using EcoRI and NotI and purified on agarose gel using the Geneclean kit. Similarly, the E3L gene was PCR-amplified with primers containing EcoRI and NotI and simultaneously digested with the same enzymes. ShuttleVacc was also double-digested with EcoRI and NotI. The NS4 and E3L ORFs were ligated into the double-digested ShuttleVacc using T4DNA ligase to generate ShuttleVacc-NS4BTV, ShuttleVacc-NS4GIV and ShuttleVacc-E3L. Recombinant shuttles were selected in XL1-Blue bacteria (Agilent Technologies, Les Ulis, France) and purified using the Qiaquick plasmid miniprep kit (Qiagen, Les Ulis, France). 

Homologous recombination between VP1080 and ShuttleVacc-NS4BTV, ShuttleVacc-NS4GIV or ShuttleVacc-E3L was performed in CEF as previously described [33]. Recombinant VP1080-BTVNS4, VP1080-GIVNS4 and VP1080-E3L were subjected to three rounds of plaque-purification and fluorescent plaques were recovered and further propagated in CEF. The expression of NS4 was confirmed by western blot analysis of lysates infected with VP1080-BTVNS4 or VP1080-GIVNS4 using antibodies to NS4, as described previously [11]. 

Monolayers of L929 cells grown in 48-well plates were pre-treated overnight with 100 units of mouse IFN α/β (Lee Biomolecular Research, San Diego, CA, USA). Cells were infected with wtVVC, VP1080, VP1080-E3L, VP1080-BTVNS4 or VP1080-GIVNS4. At 24 h post-infection, cells were harvested by scraping and total nucleic acids were extracted from the cell pellet using a ‘High pure viral nucleic acid’ kit (Roche, Basel, Switzerland). The nucleic acids were used for direct PCR assays or for the reverse transcription of RNA followed by PCR. The replication of VV in interferon-treated and untreated cells was assessed by real-time PCR, using previously described primers [44] VACV_forward/VACV_reverse and probe VACV_Probe (Table S3). Results were normalised using a eukaryotic 18S rRNA endogenous control (FAM™/MGB probe, non-primer limited) and/or Human GAPD (GAPDH) Endogenous Control (VIC^®^/MGB probe, primer limited) (Thermo Fisher Scientific, Waltham, MA, USA). 

### 2.9. Rescue of the Interferon-Sensitive Phenotype of EMCV and Replication of a ∆E3L VV Expressing NS4 in Interferon-Treated Cells

Monolayers of mouse L929 cells were pre-treated with 20 international units of mouse IFNα and IFNβ (Lee Biomolecular Research,, San Diego, CA, USA) per mL of cell culture medium in a 12-well plate. After 24 h, cells were washed with PBS and infected with wild-type or recombinant VV at a multiplicity of infection (MOI) of 1 for 4 h. Cells were washed with PBS, treated with actinomycin D (Sigma, St. Louis, MI, USA, 5 µg/mL final concentration, preventing further replication of VV) and infected with EMCV at a MOI of 10 for 1 h. Cells were washed with PBS and overlaid with medium containing 5 µg/mL actinomycin D. After 12 h or 48 h, RNA was extracted and levels of EMCV RNA assessed by real-time PCR using primers EMCVBS2/EMCVBR2 (Table S3) [34]; the results were normalized to 18S rRNA.

### 2.10. Assessing Activation of Caspases in Cells Transfected with pCIBTV1NS4 by Immunofluorescence Analysis

BSR cells were grown in 12-well plates containing cover slides. Each well was seeded with 3 × 10^5^ cells and, after 16 h, the cells were transfected with plasmid pCIBTV1NS4 and assessed for the activation of caspases using a FAM FLICA™ polycaspase kit (Bio-Rad, Hercules, CA, USA), which stains live cells, and confocal fluorescence microscopy, as recommended by the manufacturer. Briefly, the FAM-FLICA reagent was added directly to the culture medium in the wells 24 h post-transfection and incubated for 1 h at 37 °C. The cells were washed 3 times by incubating with the 1X Apoptosis Wash Buffer for 10 min at 37 °C, then fixed with ICT’s Fixative (ICT636, Immunochemistry technologies, Davis, CA, USA) and permeabilised using Triton-X100. Cells were probed with rabbit anti-NS4 antibodies (Table S2), followed by anti-rabbit Alexa Fluor 568-conjugated IgG, and stained with DAPI. Mock-transfected control cells were subjected to the same analysis.

In a separate experiment, BSR cells transfected with pCIBTV1NS4 were also assessed by fluorescence microscopy using rabbit anti-NS4 antibodies followed by anti-rabbit Alexa Fluor 488-conjugated IgG and a mouse anti-caspase 3 antibody (Table S2, Santa-Cruz Biotechnologies, Dallas, TX, USA), followed by anti-mouse Alexa Fluor 568-conjugated IgG. Nuclei were stained with DAPI. Mock-transfected cells were subjected to the same treatment and analysis. 

### 2.11. Interaction of NS4 with Caspase 3

BSR cells were grown in 12-well plates (10^5^ cells/well), then transfected with plasmids pCIBTV1NS4-6xHis (1 µg each) after 24 h, using Lipofectamine 3000 (Thermo Fisher Scientific, Waltham, MA, USA) as described by the manufacturer. At 48 h post-transfection, cells were harvested, pelleted at 500× *g* and washed twice with ice-cold PBS at 4 °C. Cell pellets were treated with RIPA buffer (50 mM Tris-HCl pH 8.0, 150 mM NaCl, 10% NP-40, 1% sodium deoxycholate, 0.1% SDS) containing EDTA-free antiprotease cocktail (Roche, Basel, Switzerland) for 30 min with a continuous rotation. The lysates were centrifuged at 13,000× *g* for 10 min at 4 °C. Clarified lysates were kept on ice and used for the pull-down of the NS4-cellular protein complexes. 

For pull-down, the Dynabeads^®^ His-Tag Isolation and Pulldown kit (Thermo Fisher Scientific, Waltham, MA, USA) was used as described by the manufacturer. Briefly, 50 µL of magnetic bead suspension was transferred into a 1.5 mL microcentrifuge tube. The ethanol bead-storage solution was removed after incubating the tube on the magnet for 2 min. The beads were washed 4 times with 300 μL 1X binding/wash buffer. Clarified transfected-cell lysates were diluted (1:1) in 2X pulldown buffer (6.5 mM sodium phosphate buffer, pH 7.4, 140 mM NaCl, 0.02% Tween-20), then mixed with the beads and incubated with rotation at 4 °C for 10 min. The magnetic beads were then pulled magnetically, the supernatant discarded and the beads were then washed 4 times with 300 μL 1X binding/wash buffer. NS4/host-cell protein–protein complexes were eluted by applying 100 µL elution buffer (300 mM imidazole, 50 mM sodium-phosphate pH 8.0, 300 mM NaCl, 0.01% Tween-20) to the beads. The elution was repeated twice more. Eluted protein complexes were analysed by 10% SDS-PAGE and western blotting using mouse anti-caspase 3 antibodies (this antibody was raised against human caspase, Santa-Cruz Biotechnologies, Dallas, TX, USA). Human and golden hamster caspase 3 amino acid sequences are 86% identical. 

### 2.12. Replication of BTV-1RG_C7_ and BTV-1∆NS4 in Cultured Mammalian Cells

HeLa or BSR cells were used to seed 24-well plates (1 × 10^5^ cells/well), in DMEM with 1% FBS. The cells were infected with BTV-1RG_C7_ or BTV-1∆NS4 at a MOI of 0.1 plaque-forming units (pfu)/cell. 

At 48 h p.i., cells were harvested and pelleted by centrifugation at 2000× *g* for 10 min at 4 °C. Cells were suspended in 2 mL of 18 MΩ water and subjected to 10 strokes in a Dounce homogeniser, in order to assist cell lysis. Lysates were transferred into a 50 mL centrifuge tube and supplemented with 8 mL of serum-free DMEM. A 10 mL aliquot of Vertrel XF (Sigma, St. Louis, MI, USA) was added and the mixture vigorously shaken in order to dissociate virus particles from cell debris [38]. Tubes were centrifuged at 2000× *g* for 10 min at 4 °C. The supernatant was collected and used for virus titrations by plaque assay in BSR cells as previously described [36].

### 2.13. Metabolic Labelling of BSR Cells Infected with BTV-1RGC7 and BTV-1∆NS4 Using L-azidohomoalanine

To assess the effect on NS4 on host-cell protein synthesis, BSR cells were infected with BTV-1RG_C7_ or BTV-1∆NS4 and subjected to pulse/chase metabolic labelling.

Metabolic labelling in infected BSR cells was performed using the Click-iT^TM^ methodology (Thermo Fisher Scientific, Waltham, MA, USA). BSR cells were plated in 24 well plates (1 × 10^5^ cells/well), 24 h prior to infection. Cells were incubated for 2 h in RPMI medium then infected by addition of BTV-1RG_C7_ or BTV-1∆NS4 at a MOI of 0.5 pfu/cell. After 1 h, excess virus was removed and cell monolayers washed twice with fresh RPMI, then supplemented with fresh RPMI containing 2% FBS. At 4 h or 8 h post-infection, cells were washed with methionine-free RPMI (Thermo Fisher Scientific, Waltham, MA, USA) and incubated in methionine-free RPMI for 30 min. Fresh methionine-free RPMI containing L-azidohomoalanine (an azido moiety-containing a methionine analogue, Thermo Fisher Scientific, Waltham, MA, USA) was added as the label. At 5 or 9 h p.i., cells were washed with PBS, then treated with 0.05% trypsin in PBS for 5 min at room temperature. RNA was extracted from a fraction of the cells (4 × 10^4^ cells) using TRIzol (Thermo Fisher Scientific, Waltham, MA, USA), treated with 10 units of RNase-free DNase I (Roche, Basel, Switzerland), then subjected to purification using RNeasy columns (Qiagen, Les Ulis, France). Another fraction of the cells (4 × 10^4^ cells) was washed with cold PBS, lysed and prepared for reaction with a biotin alkyne (alkyne and azido moieties react together generating covalently linked biotins) using the Click-iT™ protein reaction buffer kit (Thermo Fisher Scientific, Waltham, MA, USA) as described by the manufacturer. Reacted lysates were then analysed using 10% SDS-PAGE and western blot.

PCR primers CoxIHamFor/CoxIHamrev (Table S3) were designed targeting the cytochrome oxidase I gene of golden hamster (accession number: NC_013276—amplicon size = 156bp). RNA extracts from the L-azidohomoalanine-labelled cells were subjected to reverse transcription using Superscript III reverse transcriptase (Thermo Fisher Scientific, Waltham, MA, USA) and random hexaprimers. The cDNA generated was tested by real-time PCR using the QuantiTect SYBR Green PCR Kit (Qiagen, Les Ulis, France), and this test was used to normalise loading volumes of the protein in cell lysates. Aliquots of 10–12 µL of these samples were analysed using 10% SDS-PAGE then transferred onto nitrocellulose membranes, which were blocked with 5% (*w*/*v*) skimmed milk in TBST. Membranes were incubated at room temperature for 1 h with streptavidin peroxidase, then washed with TBST prior to detecting labelled proteins by chemiluminescence using ECL reagent (Bio-Rad, Hercules, CA, USA).

### 2.14. Infection of Mice with BTV-1RG_C7_ or BTV-1∆NS4

IFNAR^(-/-)^ mice represent a well-established model for studying orbivirus replication, vaccinology and assessment of antiviral molecules [36,45,46,47,48,49,50]. BTV replicates in these mice causing clinical signs. We initially assessed the virulence of our BTV-1RG_C7_ using 10, 100 or 1000 pfu/mouse in 3 groups of 5 IFNAR^(-/-)^ mice. Although clinical signs were observed by day 4, all mice recovered by day 6–7 [51], showing that BTV-1RG_C7_ is not as virulent as other BTV strains tested in this model [36,47,48]. Two groups of 5 IFNAR^(-/-)^ mice each were used to compare the effects of infection with either BTV-1RG_C7_ or BTV-1∆NS4. Blood was collected from the retro-orbital sinus on days 0, 4 and 7. RNA was extracted from the blood samples using TRIzol as previously described [36].

### 2.15. Assessment of NS4 as a Viral Suppressor of RNA Silencing (VSR)

A β-galactosidase-based reporter assay was previously developed to assess the potential function of proteins as viral suppressors of RNA silencing (VSR) [34]. We used this reporter assay to investigate whether NS4 of GIV or BTV could rescue β-galactosidase expression in cells transfected with two siRNAs (5′-GUGGAUGAAGCCAAUAUUGTT-3′ and 5′-CUAUCCCAUUACGGUCAAUTT-3′: TT are overhangs at the 3′ ends) targeting β-galactosidase mRNA. The pCIGIVNS4, pCIBTV1NS4 or pCIP19-6xHis plasmids were transfected into BSR cells 24 h prior to transfection with a mixture of the two siRNAs. A full 12-well plate of BSR cells was transfected with pCIP19-6xHis, and after 48 h the cells were collected and used to confirm expression of P19 by pull-down using a Dynabeads^®^ His-Tag Isolation and Pulldown kit as described above. The resulting protein was analysed using SDS-PAGE and Coomassie blue staining.

The previously constructed plasmid pCI-β-galactosidase (expressing the β-galactosidase enzyme under the control of the CMV promoter) [34] was used to transfect BSR cells in 12-well plates then incubated for 24 h before assaying for β-galactosidase activity in triplicate. Three wells corresponding to each NS4 were pooled and split into two fractions. RNA from one fraction was extracted with TRIzol^®^. The RNA pellet was suspended in 100 µL of RNAse-free water, treated with 1 unit of RNase-free DNAse I (Roche, Basel, Switzerland) at 37 °C for 30 min. DNAse I was inactivated by heating at 99 °C for 2 min and the RNA was back-extracted with TRIzol and resuspended in 100 µL of water. To convert RNA into cDNA, 9 µL of the RNA solution was used with SuperScript III in presence of hexaprimers, prior to real-time PCR using real-time primers and probes (at 300 nM each) specific for the NS4 mRNAs of GIV or BTV (Table S3). The other fraction was treated with the M-PER mammalian protein extraction reagent, as directed by the manufacturer, to monitor the expression of β-galactosidase using X-Gal. Protein extracts were incubated with the staining solution at 37 °C for 4 h until the positive control (containing only pCI-β-galactosidase plasmid) showed a deep blue colour. Colour development was assessed by measuring OD at 635 nm.

### 2.16. Replication of SCRV in HEK293 Cells Transduced with BTV NS4

We identified SCRV in 1999 in the *I. scapularis* tick cell line IDE2 [8] and the virus was later detected in three other tick cell lines, two *Rhipicephalus appendiculatus* cell lines and *I. scapularis*-derived IDE8 [10]. The virus failed to replicate in mammalian cells following incubation with lysates of IDE2 cells or virus purified on sucrose gradients, as previously described [8,52,53]. 

The ORF of SCRV NS4 is interrupted by an in-frame TGA stop codon (Figure S1) [11,34]. Restoring the coding capacity of this codon, to encode an arginine, generates a full-length SCRV NS4. We compared the secondary structures of SCRV NS4 (non-arbovirus) to those of BTV, GIV or AHSV (arboviruses) in an attempt to understand the mechanisms underpinning the failure of SCRV replication in mammalian cells. Analysis of the secondary structures was performed using the Chou & Fassman secondary structure prediction server (http://www.biogem.org/tool/chou-fasman/index.php (accessed on 25 August 2022)). Models for NS4 folds were generated using Phyre2 (protein homology/analogy recognition engine V 2.0) server (http://www.sbg.bio.ic.ac.uk/phyre2 (accessed on 10 March 2023)).

Because NS4 of BTV inhibits the innate immune response, we assessed the effect of BTV NS4 on replication of SCRV in HEK293 cells. This assessment was performed to understand whether SCRV’s replication in mammalian cells is restricted due to the failure of this virus to counter the mammalian innate responses. We transduced HEK293 cells with recombinant expressed TAT-NS4BTV1-6xHis, TAT-HA-VP3BTV1-6xHis of BTV (control), or full-length TAT-HA-NS4SCRV-6xHis. The presence of the TAT-tagged protein within the cells was confirmed by immunofluorescence using anti-NS4 or anti-HA tag antibodies. For these experiments, SCRV was purified from lysates of IDE2 cells as described previously [8,52,53]. ISVPs of SCRV were generated by treatment with chymotrypsin (20 mg/mL) to enhance particle infectivity, then purified as previously described [52,53]. Core particles were prepared as previously described for BTV [52,53]. Intact particles, ISVPs and cores were used to infect or transfect HEK293 cells (using Lipofectamine 2000), as previously described [54]. Tick-borne GIV grown in BSR cells was used as a control for these assays. Seg-9 copy numbers were detected in real time RT-PCR assays, to assess the replication levels of SCRV and GIV. Primers NS4GIVfor/NS4GIVrev and probe NS4GIVProb were used for GIV, primers SCRVFor1/SCRVRev1 and probe SCRVProbe1 for SCRV (Table S3). 

For transduction assays, TAT-NS4BTV1-6xHis was expressed as a GST-fused protein, purified as previously described [11,35], then treated with the 3C protease to cleave away the GST moiety. The resulting TAT-Tagged/6xHis-tagged protein was purified using Ni-NTA as previously described [37,55], then used to transduce HEK293 cell monolayers in 12-well plates (150 ng of proteins in 100 µL of 100 mM NaCl, 10 mM Tris-HCl, pH 7.5, 1 mM EDTA). At 24 h post-transduction, cells were assessed by immunofluorescence using anti-BTV-1NS4 antibodies, or anti-HA-tag antibodies. 

HEK293 cells were also infected or transfected with intact SCRV or GIV virus, purified chymotrypsin-treated virus or cores. The cells and viruses were incubated at 37 °C for 4 h before being washed twice with PBS and fresh culture medium added containing 2% FBS. At 36 h post-infection, cells were harvested, and total RNA was extracted from the pellets using TRIzol.

## 3. Results

### 3.1. Infection of IFNAR^(-/-)^ Mice with BTV-1RG_C7_ or BTV-1∆NS4

Two groups of five IFNAR^(-/-)^ mice were infected with BTV-1RG_C7_ (a clone of BTV-1 [32]) or BTV-1∆NS4 (an NS4 deletion mutant generated by reverse genetics described in Section 2.2., using the BTV-RG_C7_ backbone) and monitored for 7 days to assess the replication of both viruses. Although mice in groups infected with either BTV-1RG_C7_ or BTV-1∆NS4 showed mild clinical signs including scruffy fur, lacrimation and reduced mobility, all of them had recovered by day 6–7 p.i., with no further clinical signs observed. There was practically no difference in the onset and severity of clinical signs between the animals inoculated with these two viruses (Figure S2 and Table S4). Although NS4 can suppress the effects of interferon on virus replication, its apparent lack of an effect in these mice may well reflect their IFNAR^(-/-)^ status.

### 3.2. Replication of BTV-1 in Mammalian Cells and Metabolic Labelling

**Figure 1:**
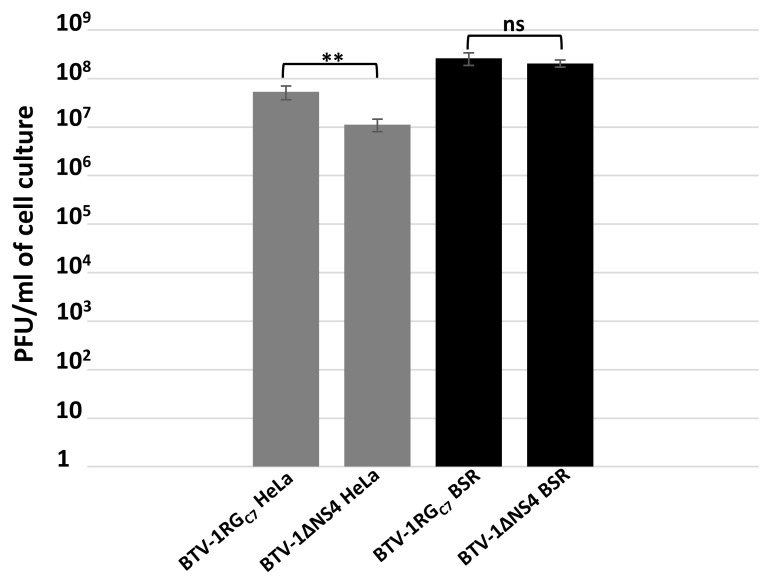
Virus titres of BTV-1RG_C7_ and BTV-1∆NS4 in BSR cells or interferon-treated HeLa cells. The cells were infected with BTV-1RG_C7_ or BTV-1∆NS4 at a MOI of 0.1 plaque-forming units (pfu)/cell. At 48 h post-infection, cells were lysed using Dounce homogenisation and virus titres determined in BSR cells, using a plaque assay. These experiments were conducted as three separate biological replicates (with 3 technical replicates each); ** = *p* < 0.01; ns = not significant.

HeLa cells are widely used to assess interferon responses [33,56,57]. BSR cells are interferon-incompetent and have a considerably weakened RIG-I (retinoic acid-inducible gene I) pathway [58,59]. Both BTV-1RG_C7_ and BTV-1∆NS4 replicate in HeLa and BSR cells (Figure 1). At 24 h p.i., virus titres in interferon-treated HeLa cells infected with BTV-1∆NS4 (determined by plaque assay [36]) were consistently ~0.8 log lower (84% reduction, calculated as previously described [32], *p* < 0.01) than in cells infected with BTV-1RG_C7_, although virus titres in BSR cells were similar for these two viruses. These results reflect the role of NS4 as an interferon antagonist. 

**Figure 2:**
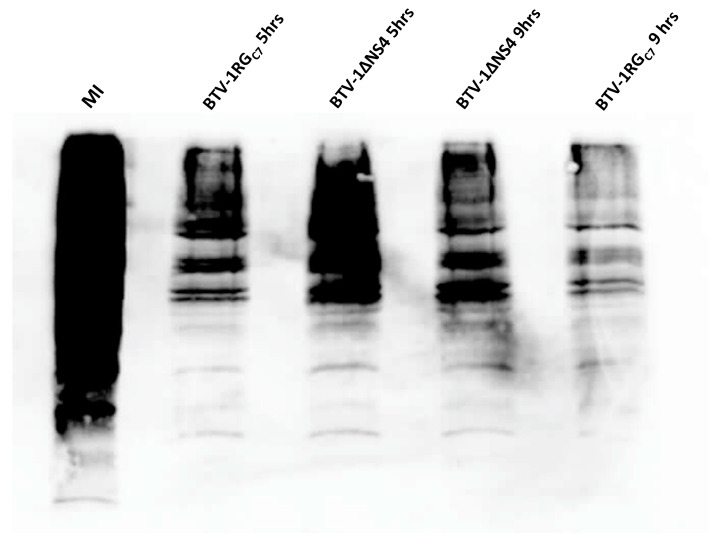
Pulse/chase metabolic labelling of BSR cells infected with BTV-1RG_C7_ or BTV-1∆NS4 at 5 h and 9 h p.i. using L-azidohomoalanine (a methionine analogue) as label. MI: mock-infected. This experiment is representative of two separate biological replicates.

Pulse/chase metabolic labelling of BSR cells infected with BTV-1RG_C7_ or BTV-1∆NS4 revealed that both viruses induce the shut-off of host cell protein synthesis (Figure 2 and Figure S3). We estimated levels of proteins in the metabolic labelling western blot assay using Bio-Rad image lab 6.1.0 tools. The results of this analysis indicate that BTV-1RG_C7_ systematically induces a higher level of shut-off than BTV-1∆NS4. At 5 or 9 h post-infection, lysates of cells infected with BTV-1RG_C7_ were found to contain 25–50% less labelled protein as compared to lysates of cells infected with BTV-1∆NS4. These results suggest that NS4 adds to the shut-off of host-cell protein synthesis, which is mainly driven by NS1 [60].

### 3.3. IFNβ Luciferase Assays

**Figure 3:**
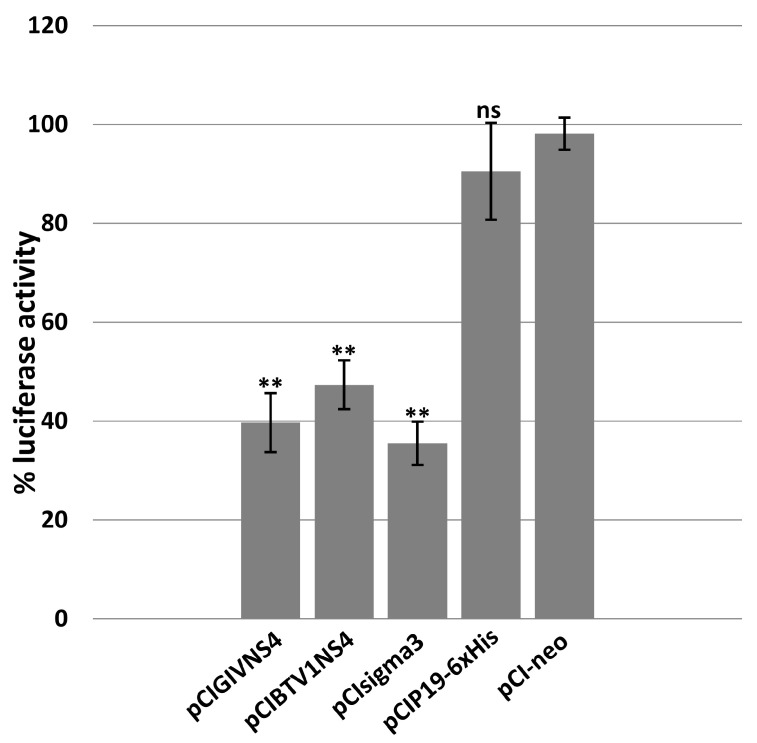
Effect of ectopically expressed NS4 on luciferase activity expressed under the control of the interferon beta promoter in HEK293 cells transfected with plasmid pGL3-IFNβ-FFLuc. GIV or BTV NS4, sigma3 of MRV, or P19 of CIRV, were expressed in HEK293 cells using the corresponding expression plasmids pCIGIVNS4, pCIBTV1NS4, pCIsigma3 or pCIP19-6xHis, respectively. The positive control consisted of lysates of HEK293 cells transfected with pGL3-IFNβ-FFLuc and pCI-neo plasmid. These experiments were conducted as triplicates (with 3 technical replicates); ** = *p* < 0.01; ns = not significant.

The roles of NS4 of BTV-1 and GIV as interferon antagonists was assessed using an IFNβ luciferase assay (Figure 3). The interferon pathway was induced by transfecting HEK293 cells with plasmid pCAGGS-Flag-2CARD-RIG-I, which expresses RIG-I CARD domains.

RIG-I, MDA5 and LPG2 (laboratory of physiology and genetics 2) are cytoplasmic pattern recognition receptors (PRRs) which recognise RNA viruses [61]. Sequences of these three proteins contain DExD/H boxes (sequence signatures associated with RNA helicases). RIG-I and MDA5 have two repeated caspase recruitment domains (CARD) located at their N-termini. dsRNA forms are selectively detected and bound by RIG-I (dsRNA < 1.5 kbp) or MDA5 (dsRNA > 3.5 kbp) exposing the CARD domain of these PRRs, activating signal transduction [62].

HEK293 cells transfected with plasmid pGL3-IFNβ-FFLuc expressing luciferase, under control of the interferon beta promoter, were used as the 100% positive control in the luciferase assay (Figure 3). The sigma3 protein of orthoreoviruses is a known antagonist of the interferon-1 pathway [63] and was used as a positive control. GIV or BTV NS4, sigma3 of MRV, or P19 of CIRV, were expressed in HEK293 cells using the corresponding expression plasmids. Expression of BTV1-NS4 or GIV-NS4 reduced the expression levels of luciferase by about 50% and 60%, respectively. These results are comparable to the reduction caused by sigma3. Cells expressing P19 of CIRV (silencing suppressor) did not cause a significant reduction in luciferase expression levels.

### 3.4. Gene Expression Assays

Innate immune pathways, including silencing, type I IFN (interferon regulatory factor (IRF) 3, 5, 7, 9, RIG-I, melanoma differentiation-associated gene 5 (MDA5), interferon beta-1 (Inf β1)), dsRNA-activated protein kinase, also known as interferon-induced protein kinase or P-68 Kinase (PKR), and viperin (also known as RSAD2), are triggered by dsRNA. Interference in several pathways by viral proteins is not unusual. Mammalian reovirus (MRV) protein σ3 (sigma3), acts as an interferon/PKR antagonist and silencing suppressor [64,65].

**Figure 4:**
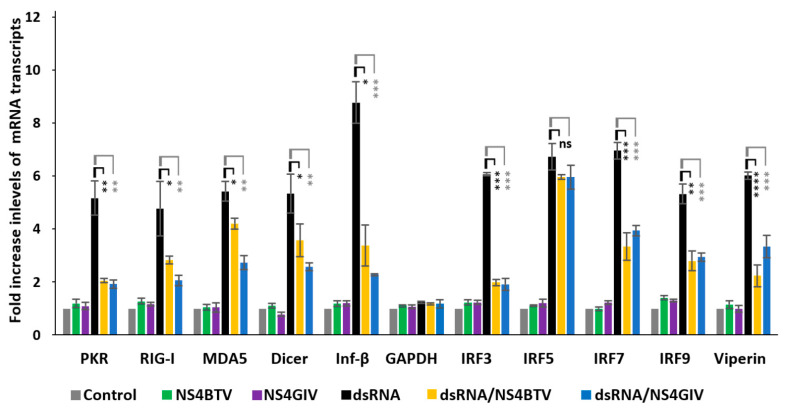
Comparison of expression of innate immune genes in HeLa cells induced by purified dsRNA from GIV-infected BSR cells (dsRNA) in the absence or presence of BTV-1 or GIV NS4. GAPDH was included as a control gene (not involved in innate immunity). These experiments were conducted as 3 separate biological replicates (with 3 technical replicates per assessed gene); * = *p*<0.05; ** = *p* < 0.01; *** = *p* < 0.001; **** = *p* < 0.0001; ns = not significant.

We transfected HeLa cells with dsRNA purified from GIV-infected BSR cells (Figure S4) to induce expression of innate immune pathways. We assessed whether NS4 expression can affect mRNA transcription of innate immunity genes listed in Table 1 (Figure 4). The ectopic expression of BTV or GIV NS4 using plasmids pCIBTV1NS4 or pCIGIVNS4 (confirmed by confocal immunofluorescence microscopy) significantly reduced the levels of these mRNA transcripts. This was corroborated by an analysis of variance (ANOVA) (performed using jamovi programme [Version 2.3.21] downloaded from https://www.jamovi.org), indicating that the reductions in transcripts of 9 out of the 10 genes tested (compared to cells without NS4) had significant *p* values (ranging from *p* < 0.05 to *p* < 0.0001, Figure 4). However, the levels of IRF5 mRNA, which were induced by dsRNA, were not significantly reduced by NS4 (Figure 4). As a control, the levels of mRNA transcripts for GAPDH were unaffected by dsRNA or NS4 expression (Figure 4). The levels of ectopic expression of NS4 mRNA (assessed by real-time PCR using NS4-specific primers and probes, Table S3) were calculated as 387 and 568 copies per cell for NS4 of BTV and GIV, respectively. The NS2 of BTV (used a negative control) did not reduce expression levels of the tested innate immune genes in cells stimulated with dsRNA (Figure S5), confirming that the reduction in transcription levels of these genes by NS4 is specific.

**Figure 5:**
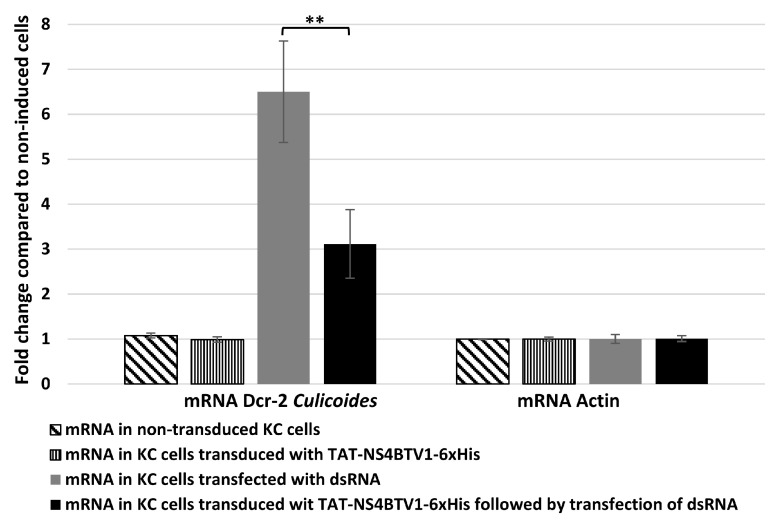
The levels of mRNA transcripts of *Culicoides* Dcr-2 and Actin-1 in untreated (non-transduced) KC cells compared with KC cells transduced with TAT-NS4BTV1-6xHis, transfected with GIV dsRNA (dsRNA), or transduced with TAT-NS4BTV1-6xHis followed by transfection with GIV dsRNA (dsRNA). Transcript expression levels were calculated using the ΔΔCt method. These experiments were conducted as two separate biological replicates (with 3 technical replicates); ** = *p* < 0.01.

Because ectopically expressed NS4 reduced gene expression levels of mammalian dicer, we assessed whether NS4 could also regulate Dicer-2 (Dcr-2) mRNA expression levels in cells of the insect vector *Culicoides*. *Culicoides* KC cells transfected with purified GIV dsRNA showed a five-fold increase in the levels Dcr-2 mRNA (Figure 5). However, in KC cells transduced with TAT-NS4-6xHis and transfected with dsRNA, the levels of Dcr-2 expression were significantly reduced (three-fold reduction, *p* < 0.01), as calculated using the ΔΔCt method (Figure 5).

### 3.5. Replication of VV Constructs and Rescue of the Interferon-Sensitive Phenotype of EMCV by Orbivirus NS4

VV replicates in interferon-treated cells and expresses genes which counteract the effects of interferon [66].

Replication of the vaccinia viruses VP1080 (an E3L-deletion mutant of VVC), VP1080-E3L (a VP1080 in which the E3L gene was restored by homologous recombination), VP1080-BTVNS4 and VP1080-GIVNS4 (VP1080 in which the ORF encoding NS4 of BTV or GIV has been inserted by homologous recombination) was assessed in interferon-treated L929 cells. As previously described [33], VP1080 exhibited an interferon-sensitive phenotype, with only a doubling of genome units compared to the inoculum at 24 h post-infection in the interferon-treated cells. In contrast, wild-type VV Copenhagen (wtVVC) replicated to significantly higher levels than VP1080 in interferon-treated cells, with an increase of 5 × 10^3^ genome units over inoculum. VP1080-E3L also replicated efficiently with an increase of 5.2 × 10^3^ genome units over inoculum, demonstrating the ability of E3L to restore the replication of VP1080 in interferon-treated L929 cells. VP1080-BTVNS4 and VP1080-GIVNS4 also replicated in interferon-treated L929 with an increase of 1.1 × 10^3^ and 4.5 × 10^3^ genome units over their respective inocula. These values are comparable to those observed for wtVVC or VP1080-E3L, indicating that NS4 of either BTV or GIV can restore the interferon-resistant phenotype, with similar efficiency to that shown by E3L.

Previous work conducted using wtVVC or VP1112 (a recombinant of VP1080 which expresses sigma3 of mammalian orthoreovirus (MRV)) has shown that both viruses are able to rescue the replication of EMCV in interferon-treated L929 mouse fibroblasts, while VP1080 (E3L deletion mutant) was unable to rescue EMCV replication [33]. VP1080-BTVNS4, VP1080-GIVNS4 and wtVVC expressing E3L were used in assays to attempt rescuing EMCV replication in interferon-treated cells.

**Figure 6:**
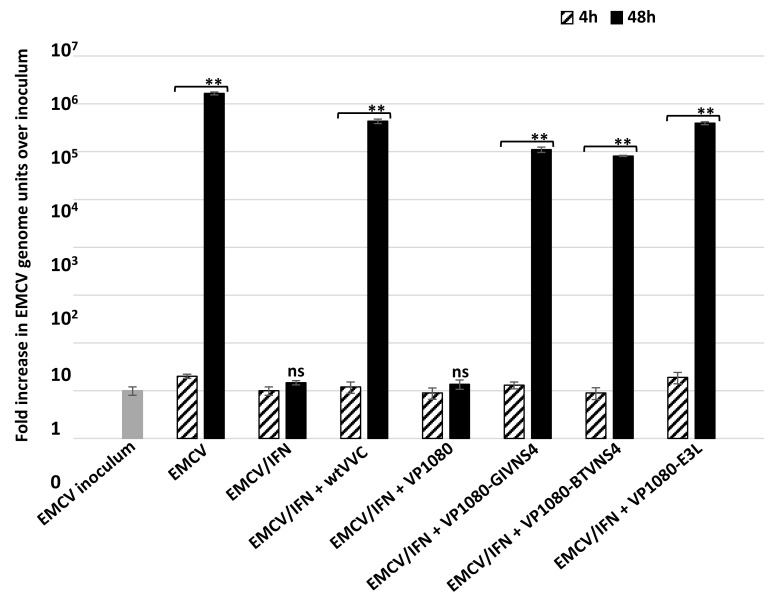
Replication of encephalomyocarditis virus (EMCV) in non-treated and interferon-treated L929 cells at 4 and 48 h post infection with EMVC alone or EMVC in co-infection with vaccinia viruses Copenhagen (wtVVC: wild-type vaccinia virus), VP1080-GIVNS4, VP1080-BTV-NS4, VP1080-E3L or VP1080. These experiments were conducted as two separate biological replicates (with 3 technical replicates); ** = *p* < 0.01; ns = not significant.

Although interferon treatment of L929 cells blocked EMCV replication, the virus did replicate in untreated cells (Figure 6). Infection of these cells with wtVVC also rescued EMCV replication, as demonstrated by an increase in EMCV genome units over the inoculum in dual-infected cells (Figure 6). Co-infection with VP1080-BTVNS4 or VP1080-GIVNS4 also supported major increases in EMCV genome units over the inoculum (3 × 10^4^, 4 × 10^4^ genome units, respectively), while infection with VP1080 generated almost no increase in EMCV replication (Figure 6). These results demonstrate that NS4 of BTV or GIV can rescue EMCV replication in interferon-treated cells, as previously shown for E3L of VV or sigma3 of MRV [33]. These findings were corroborated by the ANOVA *p* values (*p* < 0.01, Figure 6).

### 3.6. NS4 Localises with Caspase 3 in BSR Cells

NS4 of BTV was overexpressed in BSR cells by transfecting cells with pCIBTV1NS4, as confirmed by fluorescence microscopy (24 h post-transfection) using rabbit anti-NS4 antibodies and anti-rabbit Alexa Fluor 568-conjugated IgG (Figure S6).

Previous studies have shown that pro-apoptotic caspases suppress the type I interferon response by cleaving cGAS, MAVS, and IRF3 [24,25,26]. BSR transfected with pCIBTV1NS4 and expressing NS4 were stained with the FAM-FLICA™ Poly Caspase Kit (Bio-Rad, Hercules, CA, USA), which detects active caspases. The FLICA reagent binds active caspases 1, 3, 4, 5, 6, 7, 8 and 9. In BSR cells expressing NS4, active caspases were detected by the FAM-FLICA reagent (Figure S7 and z-stack Movie 1). No signal of active caspases was detected in the mock-transfected cells (Figure S7E).

**Figure 7:**
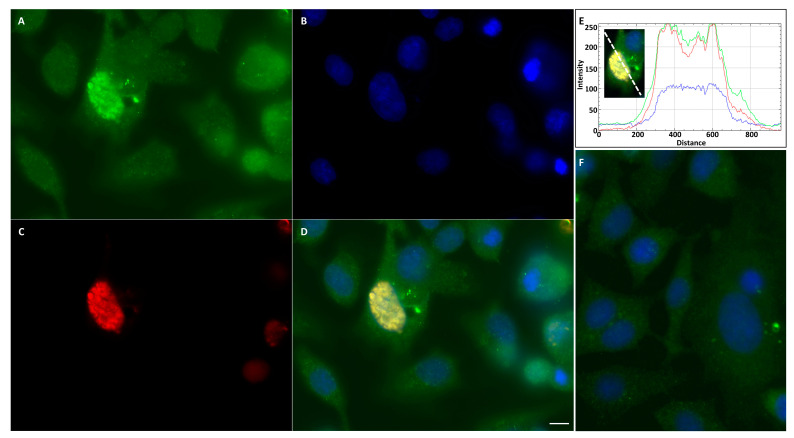
BSR cells transfected with plasmid pCIBTV1NS4 at 24 h post-transfection. (**A**): Caspase 3 stained with mouse anti-caspase 3 antibody and anti-mouse Alexa Fluor-488-conjugated IgG (green) in both the cytoplasm and nucleus of cells, (**B**): Nuclei stained with DAPI (blue), (**C**): NS4 expression detected by anti-NS4 antibodies and Alexa Fluor 568-conjugated IgG (red) and (**D**): merged (**A**–**C**) showing that NS4 localises with caspases in the nucleus. (**E**): Fluorescence intensity profiles generated in ImageJ using RGB profiler. The intensity profiles indicate that peaks of green and red fluorescence coincide. (**F**): Mock-transfected BSR cells probed with anti-caspase 3 (green) and anti-NS4 (red) antibodies. The scale bar represents 5 µm.

BSR cells transfected with pCIBTV1NS4 were also probed with anti-NS4 and anti-caspase 3 antibodies at 24 h post-transfection. This analysis indicated that NS4 localised with caspase 3 in the nucleus (Figure 7). Analysis using RGB Profiler plugin, implemented in ImageJ [67], indicated that NS4 and caspase 3 co-localised (Figure 7E), and this was corroborated by calculating Pearson’s correlation coefficient (Pearson’s coefficient = 0.9). A mock-transfected BSR cell control was probed with both anti-caspase 3 and anti-NS4 antibodies and did not identify any signal of caspase 3 associated with nuclear components (Figure 7F).

### 3.7. PolyHis Pull-Down Assay and Interaction of NS4 with Caspase 3

**Figure 8:**
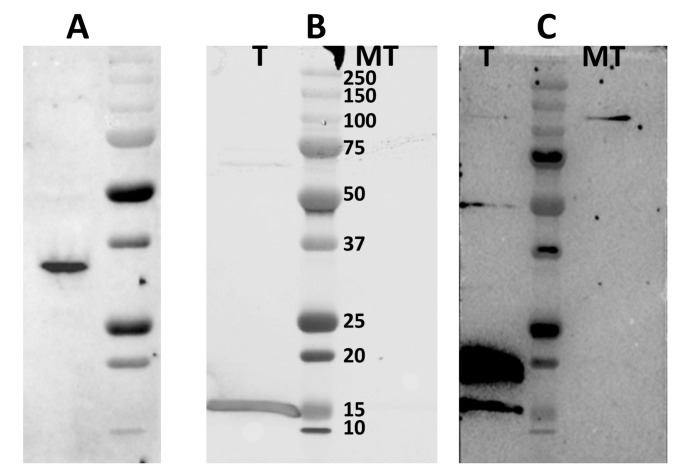
Western blot analysis detecting the protein complex of NS4 and caspase 3 in BSR cells transfected with plasmid pCIBTV1NS4-6xHis. (**A**): Western blot with mouse anti-caspase 3 antibodies detects the full-length non-cleaved caspase 3 of golden hamster (sizes ~32 kDa) in mock-transfected BSR cell lysates. Lane **T**: pull-down from BSR cells transfected with pCIBTV1NS4-6xHis, lane **MT**: pulldown from mock-transfected BSR cells. (**B**): NS4-6xHis of BTV-1 identified using western blot with rabbit anti-NS4 antibodies in complexes pulled down from lysates of BSR cells transfected with plasmid pCIBTV1NS4-6xHis. (**C**): Cleaved caspase 3 identified by anti-caspase 3 antibodies, detecting two bands of ~15 and ~17 kDa, in complexes pulled down from lysates of BSR cells transfected with plasmid pCIBTV1NS4-6xHis.

Western blot analysis using mouse anti-caspase 3 antibodies detected the full-length hamster procaspase 3 in non-transfected BSR cell lysates (Figure 8) with a size of ~32 kDa.

In order to confirm the interactions of NS4 with caspase 3, BSR cells were transfected with plasmid pCIBTV1NS4-6xHis and lysed at 48 h post-transfection, using RIPA buffer. Clarified lysates were used to pull-down proteins using nickel-coated magnetic beads to bind the 6xHis tagged NS4 protein. The pulled-down complexes were analysed by SDS-PAGE and western blotting, using mouse anti-caspase 3 antibodies (Figure 8), revealing two bands at approximately 15 and 17 kDa that were absent in the control cell lysate (Figure 8C). This indicates that NS4 interacts with the cleaved forms of caspase 3.

### 3.8. Although NS4 Downregulates Dicer Transcription, It Is Not a Conventional VSR

In a previous study [11], we showed that NS4 of GIV binds long dsRNAs, while NS4 of BTV does not. Because NS4 regulates dicer expression, we assessed whether it could also antagonise Dicer by acting as a suppressor of RNA silencing. A β-galactosidase reporter assay was used in the current study to assess the potential of NS4 from BTV-1 or GIV to act as viral-protein suppressors of RNA silencing, in comparison to the tombusvirus P19 protein, a known suppressor of RNA silencing that acts by binding siRNAs.

BSR cells were transfected with plasmids pCIGIVNS4, pCIBTV1NS4 or pCIP19-6xHis 24 h prior to transfection with a mixture of siRNAs targeting β-galactosidase. The expression of NS4 in BSR cells was confirmed using pull-down, followed by western blotting using anti-NS4 antibodies. The expression of P19 in BSR cells was also confirmed by confocal immunofluorescence and pull-down followed by Coomassie blue staining (Figure S8).

**Figure 9:**
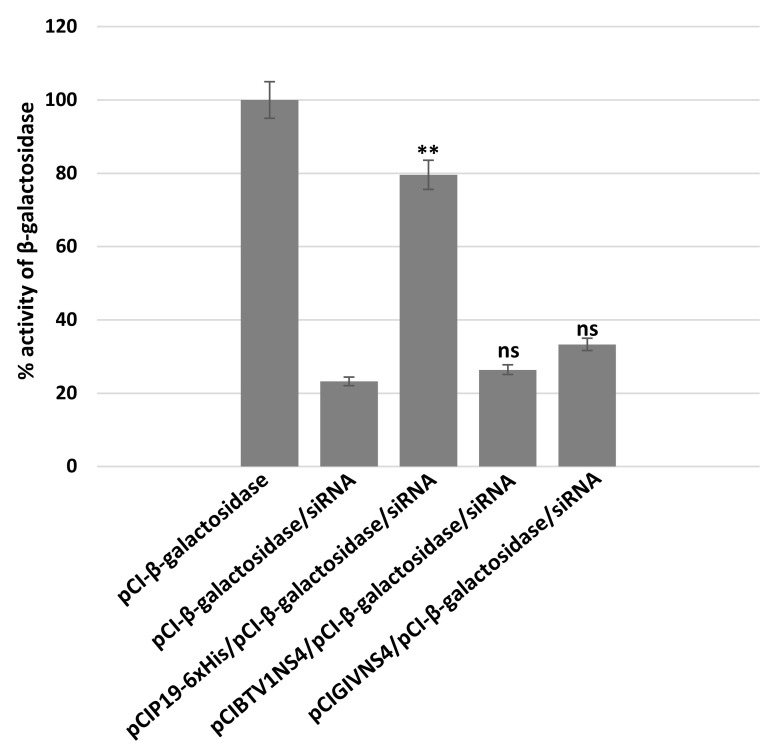
Assays of NS4 of BTV or GIV as suppressors of RNA silencing. To ectopically express BTV or GIV NS4 or CIRV P19 proteins, BSR cells were transfected with plasmids pCIGIVNS4, pCIBTV1NS4 or pCIP19-6xHis and transfected 24 h later with a mixture of siRNAs targeting β-galactosidase. Expression of β-galactosidase in BSR cells was detected by measuring optical density at 635 nm (in presence of X-Gal). These experiments were conducted as three separate biological replicates (with 3 technical replicates each); ** = *p* < 0.01; ns = not significant.

A mixture of siRNAs targeting β-galactosidase mRNA efficiently silenced the expression of β-galactosidase, reducing its expression by 77% (Figure 9). P19 rescued the expression of β-galactosidase, restoring levels to 79% (*p* < 0.01). In similar experiments, NS4 of BTV or GIV did not appear to rescue β-galactosidase expression, since its levels in cells expressing NS4 were similar to those in the silenced control (as indicated using ANOVA analysis). Therefore, despite the differences in long dsRNA binding capacity between the two NS4s, neither of them seems to bind siRNAs or act as a canonical protein suppressor of RNA silencing, as compared to P19.

### 3.9. NS4 of BTV Helps SCRV Replication in HEK293

**Figure 10:**
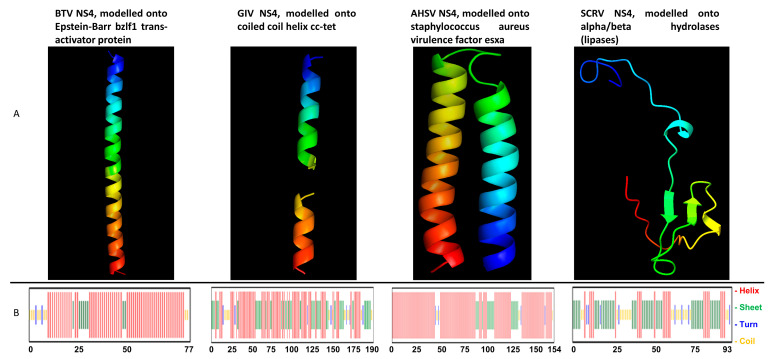
Structural models and secondary structure predictions for the NS4 proteins. (**A**): Structural models for the NS4 proteins of bluetongue virus (BTV), Great Island virus (GIV), African horse sickness virus (AHSV) and St. Croix River virus (SCRV) generated by Phyre2. (**B**): The Chou and Fassman secondary structure predictions for each of the four NS4 proteins (full-length) are shown below each of the corresponding structural models.

Modelling of NS4 using the Phyre2 programme indicated major differences between the NS4 structures of BTV, AHSV and GIV and that of SCRV (Figure 10). Chou and Fassman’s secondary structure predictions indicate that NS4 of BTV consists mainly of alpha helices (75%), with coiled coils (12%) predicted at the beginning and the end of the sequence (Figure 10). NS4 of GIV is predicted to consist mainly of alpha helices (60%) and coiled coils (11%), with the remainder predicted as turns and beat sheets. NS4 of AHSV is also predicted as mainly alpha helices (81%). For the modelling of SCRV NS4, the TGA stop codon, which interrupts the main ORF, was replaced by any one of eight possible codons, encoding six different amino acids (TCA(S), TTA(L), AGA(R), CGA(R), GGA(G), TGC(C), TGT(C) or TGG(W)) (Figure S1). The secondary structure of the resulting ‘full-length’ NS4 of SCRV was predicted to consist of 44% beta sheets, with a smaller proportion of coiled coils (22%).

BTV-1 NS4 (accession number FJ969727) was modelled in Phyre2, with levels of confidence ranging from 65% to 79%, onto transcriptional regulators such as Epstein–Barr virus (EBV) bzlf1 trans-activator protein, ccaat/enhancer-binding protein beta, also known as transcription factor c/ebp beta (CEBPB), and the general control of amino acid synthesis-like protein 4 (GCN4). The bzlf1 protein binds promoter DNA elements [68] and activates transcription. It activates two cellular stress mitogen-activated protein kinases (p38 and JNK) [69]. CEBPB is an essential transcription regulatory factor for genes which are implicated in inflammatory and immune responses [70,71,72]. GCN4 is a master transcriptional regulator that mediates the response to amino acid starvation. It binds a wide range of DNA sequences within the nucleosome or 5′ and 3′ non-coding regions [73,74,75,76,77]. The predicted secondary structure of BTV NS4 in Phyre 2 is shown in Figure S9.

GIV NS4 (accession number HM543473) was modelled onto transcriptional regulators such as *Salmonella* rep-ant protein or coiled-coil structures such as cc-tet and cc-hex2 and the chromatin structure-remodelling complex subunit rsc9.

AHSV NS4 (accession number QGY73107.1) modelled onto helical and coiled-coil proteins, such as MERS coronavirus fusion core protein, with low levels of confidence, and *Staphylococcus aureus* esxa virulence factor or mammalian DNA glycosylases. In contrast, NS4 of SCRV (accession number AF145406) modelled onto a group of functionally heterogeneous protein structures, including alpha/beta hydrolases (lipases), allophycocyanin linker chain, biotin synthase, cytochrome c oxidase, and transport proteins (Figure 10).

The secondary structure predictions and the structural models generated by Phyre2 indicate that NS4 of BTV, GIV or AHSV (arboviruses) consists mainly of helical structures while NS4 of SCRV (non-arbovirus) consists mainly of beta sheets and coils. Based on these structural differences, we tested whether NS4 of BTV would help SCRV to replicate in mammalian cells. HEK293 cells were transduced with recombinant expressed and purified TAT-NS4BTV1-6xHis, TAT-HA-VP3BTV1-6xHis or TAT-HA-NS4SCRV-6xHis (in which the stop codon was mutated to encode an arginine). Efficient delivery of these proteins was confirmed at 24 h post-transduction using immunofluorescence analysis (Figure S10).

**Figure 11:**
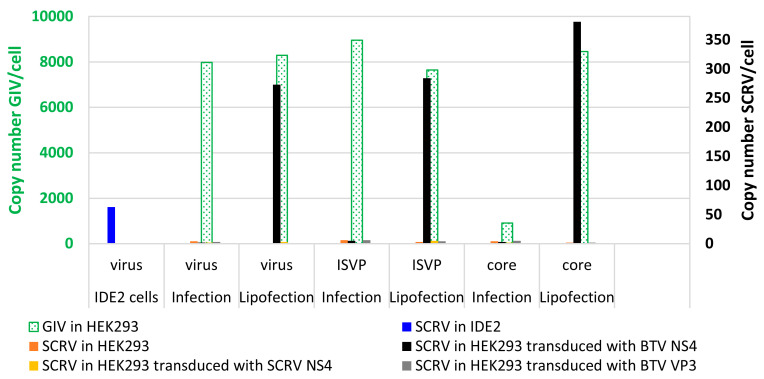
Double-axis chart showing levels of viral RNA as detected by Seg-9 real-time RT-PCR assays for SCRV or GIV in HEK293 cells. The green bars (and green axis, left) pertain to GIV RNA levels in cells infected or lipofected with various preparations of GIV used as a positive control. The black axis (right) indicates levels of SCRV RNA in cells infected or lipofected with various preparations of SCRV viral particles (virus, ISVP or core) in the presence or absence of TAT-NS4BTV1-6xHis, TAT-HA-VP3BTV1-6xHis or TAT-HA-NS4SCRV-6xHis. HEK293 cells were transduced for 24 h with TAT-NS4BTV1-6xHis, TAT-HA-VP3BTV1-6xHis or TAT-HA-NS4SCRV-6xHis, prior to infection or lipofection with virus particles. SCRV copy numbers in IDE2 tick cells are shown for reference (blue bar).

The infection of HEK293 with sucrose gradient-purified SCRV, infectious sub-viral particles, or core particles did not result in the replication of viral RNA in these cells (Figure 11). Similar results were obtained with lipofection of the same virus preparations (Figure 11). In cells transduced with VP3 of BTV as a control, or those transduced with SCRV NS4, there was also no further replication of RNA (Figure 11).

In HEK293 cells transduced with NS4 of BTV, direct infection experiments with whole virus particles, infectious subviral particles (ISVP) or core particles did not result in viral RNA replication (Figure 11). However, an increase in SCRV RNA (~250 to ~350 copies of Seg-9 mRNA/cell) was detected when the same preparations were lipofected into the cells transduced with NS4 of BTV (Figure 11). This indicates a barrier to SCRV cell attachment/entry, as well as additional intracellular restrictions of SCRV replication that can be at least partially overcome by BTV-NS4 in this mammalian cell line. These levels of replication are still much lower than those detected in HEK293 cells lipofected with the typical tick-borne GIV, which yielded ~8000 to 9000 copies of mRNA/cell after the lipofection of intact virus particles or ISVPs but not cores (Figure 11). These results suggest that there are additional barriers to a more productive infection by SCRV which are not overcome by BTV NS4. After infection of HEK293 cells with preparations of intact GIV virus particles or ISVPs, further RNA replication occurred, while infection with core particles yielded only very low levels of RNA replication. Core particles of other orbiviruses (e.g., BTV) are known to be poorly infectious for mammalian cells, although infection rates can be increased by lipofection [52,53].

## 4. Discussion

Viral non-structural proteins play important roles in virus replication. In viruses such as the alphaviruses and flaviviruses, non-structural proteins have enzymatic functions such as proteases, polymerases or capping enzymes for the viral mRNAs. Other viral non-structural proteins play important roles in intracellular trafficking, the modulation of innate immune responses, virus release from infected cells, packaging and other cell-dependent processes [78,79,80,81,82,83,84,85,86,87,88].

Orbivirus non-structural proteins are multifunctional. Some of these proteins are indispensable for virus replication, such as NS1, which acts as a positive regulator for viral RNA translation [60] and is involved in the transport of virus particles to the cell membrane. Other essential orbivirus non-structural proteins include NS2, which forms VIBs in infected cells, representing the primary site for virus assembly and with a suggested role in binding of viral mRNAs and their selection/packaging [89,90].

The mutation or deletion of arbovirus non-structural proteins can reduce replication in an animal model but does not always block replication in cell culture [91,92]. A BTV ΔNS3 deletion mutant was able to replicate in cell culture, suggesting that NS3 is non-essential, although growth in mammalian or insect cells was delayed and exit from infected insect cells was reduced [93].

Previous studies have shown that NS4 of BTV is not essential for replication, whether in cell culture or in the IFNAR^(-/-)^ mouse model [15]. Our previous studies [11] showed that NS4 localised mainly to lipid droplets in the cytoplasm, to nucleoli in the nucleus and in the cell membranes during late stages of infection with BTV. These results suggest that NS4 appears to be a multifunctional protein and its role(s) have not been fully characterised. Though NS4 of AHSV is found in the cytoplasm and nucleus of infected cells, it does not localise with the nucleoli [94]. NS4 of AHSV is an important virulence factor that suppresses host innate immunity during the early stages of infection by interfering with the JAK-STAT pathway and blocking the nuclear accumulation of STAT1 [95]. NS4 of BTV was found to counter the interferon response in ovine cells treated with interferon Tau or bovine cells treated with universal interferon [15]. In addition, NS4 of BTV was found to act in tandem with NS3, antagonising the interferon type I response by targeting STAT1 [96]. Recent analyses of the protein–protein interactions of NS4, using a yeast-two hybrid screen, identified NS4 interactions with multiple cellular proteins [97].

The aim of the current study was to investigate functions of the orbivirus NS4 and our findings indicate multiple additional roles for this protein.

Viruses such as EMCV and vesicular stomatitis virus fail to replicate in cells treated with interferon. The mediator of this inhibition was previously identified as a 40 kDa enzyme known as 2′-5′-oligoadenylate synthetase (OAS) [98]. The known function of OAS is the activation of RNase L, a latent endoribonuclease [99]. RNase L is an essential component of innate immune mechanisms, cleaving single-stranded loops of mRNA (viral or cellular), generating small RNAs with a 5′-OH and 3′-monophosphate [100]. The ssRNAs of viruses are cleaved by RNase L into small RNAs known as suppressors of virus RNA (because they activate RIG-I and inhibit virus replication) [101,102]. OAS converts ATP into 2′-5′-oligoadenylate, which accumulates in interferon-treated EMCV-infected cells. A dsRNA intermediate of EMCV replication is produced in virus-infected cells. This intermediate activates OAS in vitro. In cells infected with EMCV, MDA5 triggers innate immune signalling [103]. EMCV, on the other hand, degrades the RIG-I sensor in infected cells [56]. EMCV is unable to replicate in interferon-treated cells because interferon induces the activation of PKR. E3L is known to inhibit the activation of PKR by interferon, thereby rescuing the interferon-sensitive phenotype of EMCV during a co-infection with wtVVC [33]. We showed that NS4 of BTV can rescue replication of an E3L-defective VV or EMCV in mouse fibroblasts treated with mouse interferon, confirming a role for NS4 in suppressing the effects of interferon.

Our results show that NS4 inhibits the human interferon beta promoter (driving expression of a firefly luciferase in a reporter assay) with comparable efficiency to MRV3 sigma3 protein, a well-known inhibitor of type I interferon. E3L and sigma3 are both dsRNA-binding proteins and were characterised as inhibitors of interferon type I, PKR, OAS and RNA silencing [33,63,104,105,106].

Our results indicate that NS4 downregulates Dicer transcription in both HeLa cells and KC cells. We also previously showed that NS4 of GIV (20 kDa), but not NS4 of BTV (9.5 kDa) binds dsRNA [11]. In addition to being an anti-interferon/PKR, E3L of vaccinia virus is also a suppressor of RNA silencing [18,19]. Because NS4 can replace E3L in vaccinia virus, we assessed in the current study whether NS4 may also act as a typical viral suppressors of RNA silencing. Our results indicate that, compared to P19 (a well-known VSR), neither of the NS4 proteins (BTV or GIV) rescued silenced β-galactosidase in a detection assay for the viral suppressors of RNA silencing. Our results showed that NS4 of GIV or BTV modulates the expression of several genes, including interferon and other interferon-stimulated genes, in cells where pathways are induced by dsRNA.

BTV activates both extrinsic and intrinsic pathways of apoptosis by activating caspases 3, 7, 8 and 9 [16]. Although NF-κB is activated in BTV-infected cells [16,107], it has been shown that it is unlikely responsible for apoptosis, which appears to be principally linked to the degradation of Iκbα [16]. A recent study of BTV NS4 protein–protein interaction cartography identified multiple interacting cellular-proteins, including apoptosis antagonising factor (AATF) [97]. AATF is a known anti-apoptotic factor that mediates its anti-apoptotic effect by the transcriptional regulation of AKT1 [108] or repression of p53-mediated apoptosis [109]. In the current study, we have shown that in cells transfected with plasmid pCIBTV1NS4, BTV-1 NS4 localised with active caspases in the nuclei, as detected using the FAM-FLICA assay and anti-caspase 3 antibodies. This was corroborated by a pull-down assay performed using lysates of cells transfected with pCIBTV1NS4-6xHis. NS4 was identified together with cleaved caspase 3 in the pull-down protein complexes. The type I interferon response can be suppressed by pro-apoptotic caspases, as previously shown [24,25,26]. Our findings show that NS4 interacts with active caspase 3, and by doing so it may regulate the apoptotic process and/or likely contribute to dampening the interferon type I response.

SCRV is the only known arthropod-only (tick-only) orbivirus to date. We previously reported that the ORF of SCRV NS4 is interrupted by a stop codon, thus preventing the expression of a full-length NS4 [8,11]. SCRV replicates in tick cells, causing long-term persistent infection, but does not infect mammalian cells [9]. Transducing mammalian cells with a modified ‘full-length-NS4′ of SCRV did not have any effect on replication following infection/lipofection of mammalian cells with SCRV. However, when the same cells were transduced with BTV NS4, we observed an increase in levels of transcribed SCRV RNA, suggesting that BTV NS4 helps SCRV to partially overcome intracellular blocks to its replication, likely by dampening the mammalian innate immune responses.

## 5. Conclusions

We conclude that NS4 of mammalian orbiviruses is involved in modulating the cellular innate responses, particularly in mammalian cells. NS4 dampens the interferon response and interacts with pro-apoptotic caspases (particularly caspase 3), playing an additional role as a virulence factor in pathogenesis.

## Figures and Tables

**Table 1:** Proprietary real-time PCR assays (all probe-based) from Thermo Fisher Scientific (Applied Biosystems) used in assessing expression of various cellular genes.

Gene Name	Assay ID
Eukaryotic 18S rRNA	Hs99999901_s1
IRF 3	Hs01547283_m1
IRF 5	Hs00158114_m1
IRF 9	Hs00196051_m1
IRF 7	Hs01014809_g1
PKR	Hs00169345_m1
Dicer	Hs00229023_m1
MDA5	Hs01070332_m1
Interferon β1	Hs01077958_s1
GAPDH	Hs03929097_g1
RIG-I	Hs00204833_m1
RSAD2 (viperin)	Hs02265339_cn

IRF: interferon regulatory factor; GAPDH: Glyceraldehyde 3-phosphate dehydrogenase; MDA5: Melanoma differentiation-associated protein 5; RIG-I: retinoic acid inducible gene I.

## Data Availability

All data are presented in the main manuscript and supporting supplementary material.

## Supplementary Materials

The following supporting information can be downloaded at: https://www.mdpi.com/article/10.3390/v15091908/s1, Table S1: Primer sequences used for cloning into mammalian and bacterial expression plasmids, Table S2: Antibodies used in immunofluorescence and western blot analyses, Table S3: Primer sequences used for real time PCRs or cloning, Table S4: Ct values and virus titres in blood of mice infected with BTV-1RG_C7_ or BTV-1∆NS4 at day 4 post-infection (p.i.), Figure S1: Sequence of genome segment 9 of St Croix River virus, Figure S2: Ct values for viral RNAemia, determined using RT-PCR, and virus titres expressed in pfu/mL, determined by plaque assay, in mice inoculated with BTV-1RG_C7_ or BTV-1∆NS4, Figure S3: Pulse/chase metabolic labelling of BSR cells infected with BTV-1RGC7 or BTV-1∆NS4, Figure S4: Purified dsRNA of Great Island virus analysed by polyacrylamide gel electrophoresis, Figure S5: Comparison of expression of innate immune genes in HeLa cells induced by purified dsRNA from GIV-infected BSR cells (dsRNA) in the absence or presence of BTV-1 NS2, Figure S6: BSR cells transfected with plasmid pCIBTV1NS4 (at 24 h post-transfection). Figure S7: BSR cells transfected with plasmid pCIBTV1NS4 and assessed by FAM-FLICA at 24 h post-transfection, Figure S8: Expression of CIRV P19 of in BSR cells transfected with plasmid pCIP19-6xHis, Figure S9: Secondary structure predictions for the amino acid sequence of BTV NS4 using Phyre2, Figure S10: HEK293 cells transduced with TAT-tagged proteins.

## References

[1] Attoui H., Mohd Jaafar F., Mertens P.P.C. The Reovirales a new taxonomic order: Families Sedoreoviridae and Spinareoviridae. Presented at the 13th International dsRNA Virus Symposium.

[2] Matthijnssens J., Attoui H., Banyai K., Brussaard C.P.D., Danthi P., Del Vas M., Dermody T.S., Duncan R., Fang Q., Johne R. (2022). ICTV Virus Taxonomy Profile: *Sedoreoviridae* 2022. J. Gen. Virol..

[3] Mohd Jaafar F., Belhouchet M., Belaganahalli M., Tesh R.B., Mertens P.P., Attoui H. (2014). Full-genome characterisation of Orungo, Lebombo and Changuinola viruses provides evidence for co-evolution of orbiviruses with their arthropod vectors. PLoS ONE.

[4] Belaganahalli M.N., Maan S., Maan N.S., Brownlie J., Tesh R., Attoui H., Mertens P.P. (2015). Genetic characterization of the tick-borne orbiviruses. Viruses.

[5] Batten C., Darpel K., Henstock M., Fay P., Veronesi E., Gubbins S., Graves S., Frost L., Oura C. (2014). Evidence for transmission of bluetongue virus serotype 26 through direct contact. PLoS ONE.

[6] Batten C.A., Henstock M.R., Steedman H.M., Waddington S., Edwards L., Oura C.A. (2013). Bluetongue virus serotype 26: Infection kinetics, pathogenesis and possible contact transmission in goats. Vet. Microbiol..

[7] Attoui H., Mertens P.P.C., Becnel J., Belaganahalli M., Bergoin M., Brussaard C.P., Chappell J.D., Ciarlet M., del Vas M., Dermody T.S., King A.M.Q., Adams M.J., Carstens E.B., Lefkowitz E.J. (2012). Reoviridae. Virus Taxonomy. The Ninth Report of the International Committee on Taxonomy of Viruses.

[8] Attoui H., Stirling J.M., Munderloh U.G., Billoir F., Brookes S.M., Burroughs J.N., de Micco P., Mertens P.P.C., de Lamballerie X. (2001). Complete sequence characterization of the genome of the St Croix River virus, a new orbivirus isolated from cells of Ixodes scapularis. J. Gen. Virol..

[9] Bell-Sakyi L., Attoui H. (2013). Endogenous tick viruses and modulation of tick-borne pathogen growth. Front. Cell Infect. Microbiol..

[10] Alberdi M.P., Dalby M.J., Rodriguez-Andres J., Fazakerley J.K., Kohl A., Bell-Sakyi L. (2012). Detection and identification of putative bacterial endosymbionts and endogenous viruses in tick cell lines. Ticks Tick. Borne Dis..

[11] Belhouchet M., Mohd Jaafar F., Firth A.E., Grimes J.M., Mertens P.P., Attoui H. (2011). Detection of a fourth orbivirus non-structural protein. PLoS ONE.

[12] Mertens P.P., Brown F., Sangar D.V. (1984). Assignment of the genome segments of bluetongue virus type 1 to the proteins which they encode. Virology.

[13] Mohd Jaafar F., Monsion B., Mertens P.P.C., Attoui H. (2023). Identification of Orbivirus Non-Structural Protein 5 (NS5), Its Role and Interaction with RNA/DNA in Infected Cells. Int. J. Mol. Sci..

[14] Belhouchet M., Mohd Jaafar F., Tesh R., Grimes J., Maan S., Mertens P.P., Attoui H. (2010). Complete sequence of Great Island virus and comparison with the T2 and outer-capsid proteins of Kemerovo, Lipovnik and Tribec viruses (genus Orbivirus, family Reoviridae). J. Gen. Virol..

[15] Ratinier M., Caporale M., Golder M., Franzoni G., Allan K., Nunes S.F., Armezzani A., Bayoumy A., Rixon F., Shaw A. (2011). Identification and characterization of a novel non-structural protein of bluetongue virus. PLoS Pathog..

[16] Stewart M.E., Roy P. (2010). Role of cellular caspases, nuclear factor-kappa B and interferon regulatory factors in Bluetongue virus infection and cell fate. Virol. J..

[17] Firth A.E. (2008). Bioinformatic analysis suggests that the Orbivirus VP6 cistron encodes an overlapping gene. Virol. J..

[18] Li F., Ding S.W. (2006). Virus counterdefense: Diverse strategies for evading the RNA-silencing immunity. Annu. Rev. Microbiol..

[19] Li W.X., Li H., Lu R., Li F., Dus M., Atkinson P., Brydon E.W., Johnson K.L., Garcia-Sastre A., Ball L.A. (2004). Interferon antagonist proteins of influenza and vaccinia viruses are suppressors of RNA silencing. Proc. Natl. Acad. Sci. USA.

[20] Chawla-Sarkar M., Leaman D.W., Borden E.C. (2001). Preferential induction of apoptosis by interferon (IFN)-β compared with IFN-α2: Correlation with TRAIL/Apo2L induction in melanoma cell lines. Clin. Cancer Res..

[21] Steen H.C., Gamero A.M. (2010). Interferon-lambda as a potential therapeutic agent in cancer treatment. J. Interferon Cytokine Res..

[22] Lokshin A., Mayotte J.E., Levitt M.L. (1995). Mechanism of interferon beta-induced squamous differentiation and programmed cell death in human non-small-cell lung cancer cell lines. J. Natl. Cancer Inst..

[23] Rodriguez-Villanueva J., McDonnell T.J. (1995). Induction of apoptotic cell death in non-melanoma skin cancer by interferon-alpha. Int. J. Cancer.

[24] Ning X., Wang Y., Jing M., Sha M., Lv M., Gao P., Zhang R., Huang X., Feng J.M., Jiang Z. (2019). Apoptotic Caspases Suppress Type I Interferon Production via the Cleavage of cGAS, MAVS, and IRF3. Mol. Cell.

[25] Rajput A., Kovalenko A., Bogdanov K., Yang S.H., Kang T.B., Kim J.C., Du J., Wallach D. (2011). RIG-I RNA helicase activation of IRF3 transcription factor is negatively regulated by caspase-8-mediated cleavage of the RIP1 protein. Immunity.

[26] Rongvaux A., Jackson R., Harman C.C., Li T., West A.P., de Zoete M.R., Wu Y., Yordy B., Lakhani S.A., Kuan C.Y. (2014). Apoptotic caspases prevent the induction of type I interferons by mitochondrial DNA. Cell.

[27] Sato M., Maeda N., Yoshida H., Urade M., Saito S. (1977). Plaque formation of herpes virus hominis type 2 and rubella virus in variants isolated from the colonies of BHK21/WI-2 cells formed in soft agar. Arch. Virol..

[28] Wechsler S.J., McHolland L.E., Wilson W.C. (1991). A RNA virus in cells from Culicoides variipennis. J. Invertebr. Pathol..

[29] Munderloh U.G., Liu Y., Wang M., Chen C., Kurtti T.J. (1994). Establishment, maintenance and description of cell lines from the tick Ixodes scapularis. J. Parasitol..

[30] Munderloh U.G., Kurtti T.J. (1989). Formulation of medium for tick cell culture. Exp. Appl. Acarol..

[31] Henle G., Deinhardt F., Bergs V.V., Henle W. (1958). Studies on persistent infections of tissue cultures. I. General aspects of the system. J. Exp. Med..

[32] Attoui H., Monsion B., Klonjkowski B., Zientara S., Mertens P.P.C., Mohd Jaafar F. (2021). Identification of the Genome Segments of Bluetongue Virus Type 26/Type 1 Reassortants Influencing Horizontal Transmission in a Mouse Model. Viruses.

[33] Beattie E., Denzler K.L., Tartaglia J., Perkus M.E., Paoletti E., Jacobs B.L. (1995). Reversal of the interferon-sensitive phenotype of a vaccinia virus lacking E3L by expression of the reovirus S4 gene. J. Virol..

[34] Belhouchet M. (2013). Analysis of an Anti-Silencing Mechanism Involved in Immune Evasion by Vector-Borne dsRNA Animal Viruses of Family *Reoviridae*. Ph.D. Thesis.

[35] Mohd Jaafar F., Attoui H., Gallian P., Biagini P., Cantaloube J.F., de Micco P., de Lamballerie X. (2003). Recombinant VP7-based enzyme-linked immunosorbent assay for detection of immunoglobulin G antibodies to Colorado tick fever virus. J. Clin. Microbiol..

[36] Mohd Jaafar F., Belhouchet M., Vitour D., Adam M., Breard E., Zientara S., Mertens P.P., Attoui H. (2014). Immunisation with bacterial expressed VP2 and VP5 of bluetongue virus (BTV) protect α/β interferon-receptor knock-out (IFNAR^−/−^) mice from homologous lethal challenge. Vaccine.

[37] Schwarze S.R., Hruska K.A., Dowdy S.F. (2000). Protein transduction: Unrestricted delivery into all cells?. Trends Cell Biol..

[38] Mohd Jaafar F., Attoui H., Mertens P.P.C., de Micco P., de Lamballerie X. (2005). Structural organization of an encephalitic human isolate of *Banna virus* (genus *Seadornavirus*, family Reoviridae). J. Gen. Virol..

[39] Steidle S., Martinez-Sobrido L., Mordstein M., Lienenklaus S., Garcia-Sastre A., Staheli P., Kochs G. (2010). Glycine 184 in nonstructural protein NS1 determines the virulence of influenza A virus strain PR8 without affecting the host interferon response. J. Virol..

[40] Attoui H., Billoir F., Cantaloube J.F., Biagini P., de Micco P., de Lamballerie X. (2000). Strategies for the sequence determination of viral dsRNA genomes. J. Virol. Methods.

[41] Bellamy A.R., Shapiro L., August J.T., Joklik W.K. (1967). Studies on reovirus RNA. I. Characterization of reovirus genome RNA. J. Mol. Biol..

[42] Castillo A., Cifuentes V. (1994). Presence of double-stranded RNA and virus-like particles in Phaffia rhodozyma. Curr. Genet..

[43] Attoui H., Billoir F., Bruey J.M., de Micco P., de Lamballerie X. (1998). Serologic and molecular diagnosis of Colorado tick fever viral infections. Am. J. Trop. Med. Hyg..

[44] Vanpouille C., Biancotto A., Lisco A., Brichacek B. (2007). Interactions between human immunodeficiency virus type 1 and vaccinia virus in human lymphoid tissue ex vivo. J. Virol..

[45] Alonso C., Utrilla-Trigo S., Calvo-Pinilla E., Jimenez-Cabello L., Ortego J., Nogales A. (2020). Inhibition of Orbivirus Replication by Aurintricarboxylic Acid. Int. J. Mol. Sci..

[46] Calvo-Pinilla E., de la Poza F., Gubbins S., Mertens P.P., Ortego J., Castillo-Olivares J. (2015). Antiserum from mice vaccinated with modified vaccinia *Ankara virus* expressing African horse sickness virus (AHSV) VP2 provides protection when it is administered 48h before, or 48h after challenge. Antivir. Res..

[47] Calvo-Pinilla E., Rodriguez-Calvo T., Sevilla N., Ortego J. (2009). Heterologous prime boost vaccination with DNA and recombinant modified vaccinia virus Ankara protects IFNAR^−/−^ mice against lethal bluetongue infection. Vaccine.

[48] Jabbar T.K., Calvo-Pinilla E., Mateos F., Gubbins S., Bin-Tarif A., Bachanek-Bankowska K., Alpar O., Ortego J., Takamatsu H.H., Mertens P.P. (2013). Protection of IFNAR^−/−^ mice against bluetongue virus serotype 8, by heterologous (DNA/rMVA) and homologous (rMVA/rMVA) vaccination, expressing outer-capsid protein VP2. PLoS ONE.

[49] Fay P.C., Attoui H., Batten C., Mohd Jaafar F., Lomonossoff G.P., Daly J.M., Mertens P.P.C. (2019). Bluetongue virus outer-capsid protein VP2 expressed in Nicotiana benthamiana raises neutralising antibodies and a protective immune response in IFNAR^−/−^ mice. Vaccine X.

[50] Mohd Jaafar F., Monsion B., Belhouchet M., Mertens P.P.C., Attoui H. (2021). Inhibition of Orbivirus Replication by Fluvastatin and Identification of the Key Elements of the Mevalonate Pathway Involved. Viruses.

[51] Attoui H., Mohd Jaafar F., Monsion B., Klonjkowski B., Reid E., Fay P.C., Saunders K., Lomonossoff G., Haig D., Mertens P.P.C. (2023). Increased Clinical Signs and Mortality in IFNAR^−/−^ Mice Immunised with the Bluetongue Virus Outer-Capsid Proteins VP2 or VP5, after Challenge with an Attenuated Heterologous Serotype. Pathogens.

[52] Burroughs J.N., O’Hara R.S., Smale C.J., Hamblin C., Walton A., Armstrong R., Mertens P.P. (1994). Purification and properties of virus particles, infectious subviral particles, cores and VP7 crystals of African horsesickness virus serotype 9. J. Gen. Virol..

[53] Mertens P.P., Burroughs J.N., Anderson J. (1987). Purification and properties of virus particles, infectious subviral particles, and cores of bluetongue virus serotypes 1 and 4. Virology.

[54] Hill C.L., Booth T.F., Stuart D.I., Mertens P.P. (1999). Lipofectin increases the specific activity of cypovirus particles for cultured insect cells. J. Virol. Methods.

[55] Becker-Hapak M., McAllister S.S., Dowdy S.F. (2001). TAT-mediated protein transduction into mammalian cells. Methods.

[56] Papon L., Oteiza A., Imaizumi T., Kato H., Brocchi E., Lawson T.G., Akira S., Mechti N. (2009). The viral RNA recognition sensor RIG-I is degraded during encephalomyocarditis virus (EMCV) infection. Virology.

[57] Daher A., Laraki G., Singh M., Melendez-Pena C.E., Bannwarth S., Peters A.H., Meurs E.F., Braun R.E., Patel R.C., Gatignol A. (2009). TRBP control of PACT-induced phosphorylation of protein kinase R is reversed by stress. Mol. Cell Biol..

[58] Brzozka K., Finke S., Conzelmann K.K. (2005). Identification of the rabies virus alpha/beta interferon antagonist: Phosphoprotein P interferes with phosphorylation of interferon regulatory factor 3. J. Virol..

[59] Habjan M., Penski N., Spiegel M., Weber F. (2008). T7 RNA polymerase-dependent and -independent systems for cDNA-based rescue of Rift Valley fever virus. J. Gen. Virol..

[60] Boyce M., Celma C.C., Roy P. (2012). Bluetongue virus non-structural protein 1 is a positive regulator of viral protein synthesis. Virol. J..

[61] Yoneyama M., Fujita T. (2009). RNA recognition and signal transduction by RIG-I-like receptors. Immunol. Rev..

[62] Barral P.M., Sarkar D., Su Z.Z., Barber G.N., DeSalle R., Racaniello V.R., Fisher P.B. (2009). Functions of the cytoplasmic RNA sensors RIG-I and MDA-5: Key regulators of innate immunity. Pharmacol. Ther..

[63] Jacobs B.L., Langland J.O. (1998). Reovirus sigma 3 protein: dsRNA binding and inhibition of RNA-activated protein kinase. Curr. Top. Microbiol. Immunol..

[64] Lichner Z., Silhavy D., Burgyan J. (2003). Double-stranded RNA-binding proteins could suppress RNA interference-mediated antiviral defences. J. Gen. Virol..

[65] Mérai Z., Kerényi Z., Kertész S., Magna M., Lakatos L., Silhavy D. (2006). Double-stranded RNA binding may be a general plant RNA viral strategy to suppress RNA silencing. J. Virol..

[66] Smith G.L., Talbot-Cooper C., Lu Y. (2018). How Does Vaccinia Virus Interfere With Interferon?. Adv. Virus Res..

[67] Schneider C.A., Rasband W.S., Eliceiri K.W. (2012). NIH Image to ImageJ: 25 years of image analysis. Nat. Methods.

[68] Sinclair A.J., Brimmell M., Shanahan F., Farrell P.J. (1991). Pathways of activation of the Epstein-Barr virus productive cycle. J. Virol..

[69] Adamson A.L., Darr D., Holley-Guthrie E., Johnson R.A., Mauser A., Swenson J., Kenney S. (2000). Epstein-Barr virus immediate-early proteins BZLF1 and BRLF1 activate the ATF2 transcription factor by increasing the levels of phosphorylated p38 and c-Jun N-terminal kinases. J. Virol..

[70] Kinoshita S., Akira S., Kishimoto T. (1992). A member of the C/EBP family, NF-IL6 beta, forms a heterodimer and transcriptionally synergizes with NF-IL6. Proc. Natl. Acad. Sci. USA.

[71] Roy S.K., Hu J., Meng Q., Xia Y., Shapiro P.S., Reddy S.P., Platanias L.C., Lindner D.J., Johnson P.F., Pritchard C. (2002). MEKK1 plays a critical role in activating the transcription factor C/EBP-β-dependent gene expression in response to IFN-gamma. Proc. Natl. Acad. Sci. USA.

[72] Pless O., Kowenz-Leutz E., Knoblich M., Lausen J., Beyermann M., Walsh M.J., Leutz A. (2008). G9a-mediated lysine methylation alters the function of CCAAT/enhancer-binding protein-beta. J. Biol. Chem..

[73] Rawal Y., Chereji R.V., Valabhoju V., Qiu H., Ocampo J., Clark D.J., Hinnebusch A.G. (2018). Gcn4 Binding in Coding Regions Can Activate Internal and Canonical 5′ Promoters in Yeast. Mol. Cell.

[74] Natarajan K., Meyer M.R., Jackson B.M., Slade D., Roberts C., Hinnebusch A.G., Marton M.J. (2001). Transcriptional profiling shows that Gcn4p is a master regulator of gene expression during amino acid starvation in yeast. Mol. Cell Biol..

[75] Schmidheini T., Mosch H.U., Graf R., Braus G.H. (1990). A GCN4 protein recognition element is not sufficient for GCN4-dependent regulation of transcription in the ARO7 promoter of Saccharomyces cerevisiae. Mol. Gen. Genet..

[76] Mosch H.U., Scheier B., Lahti R., Mantsala P., Braus G.H. (1991). Transcriptional activation of yeast nucleotide biosynthetic gene ADE4 by GCN4. J. Biol. Chem..

[77] Stanojevic D., Verdine G.L. (1995). Deconstruction of GCN4/GCRE into a monomeric peptide-DNA complex. Nat. Struct. Biol..

[78] Elazar M., Cheong K.H., Liu P., Greenberg H.B., Rice C.M., Glenn J.S. (2003). Amphipathic helix-dependent localization of NS5A mediates hepatitis C virus RNA replication. J. Virol..

[79] Kohl A., Clayton R.F., Weber F., Bridgen A., Randall R.E., Elliott R.M. (2003). Bunyamwera virus nonstructural protein NSs counteracts interferon regulatory factor 3-mediated induction of early cell death. J. Virol..

[80] Konan K.V., Giddings T.H., Ikeda M., Li K., Lemon S.M., Kirkegaard K. (2003). Nonstructural protein precursor NS4A/B from hepatitis C virus alters function and ultrastructure of host secretory apparatus. J. Virol..

[81] Krug R.M., Yuan W., Noah D.L., Latham A.G. (2003). Intracellular warfare between human influenza viruses and human cells: The roles of the viral NS1 protein. Virology.

[82] Liu W.J., Chen H.B., Khromykh A.A. (2003). Molecular and functional analyses of Kunjin virus infectious cDNA clones demonstrate the essential roles for NS2A in virus assembly and for a nonconservative residue in NS3 in RNA replication. J. Virol..

[83] Nibert M.L. (2002). Rotavirus translation control protein takes RNA to heart. Structure.

[84] Noah D.L., Twu K.Y., Krug R.M. (2003). Cellular antiviral responses against influenza A virus are countered at the posttranscriptional level by the viral NS1A protein via its binding to a cellular protein required for the 3′ end processing of cellular pre-mRNAS. Virology.

[85] Varani G., Allain F.H. (2002). How a rotavirus hijacks the human protein synthesis machinery. Nat. Struct. Biol..

[86] Young S., Cordy D.R. (1964). An Ovine Fetal Encephalopathy Caused by Bluetongue Vaccine Virus. J. Neuropathol. Exp. Neurol..

[87] Zhirnov O.P., Konakova T.E., Wolff T., Klenk H.D. (2002). NS1 protein of influenza A virus down-regulates apoptosis. J. Virol..

[88] Owens R.J., Limn C., Roy P. (2004). Role of an arbovirus nonstructural protein in cellular pathogenesis and virus release. J. Virol..

[89] Mumtsidu E., Makhov A.M., Roessle M., Bathke A., Tucker P.A. (2007). Structural features of the Bluetongue virus NS2 protein. J. Struct. Biol..

[90] Zhao Y., Thomas C., Bremer C., Roy P. (1994). Deletion and mutational analyses of bluetongue virus NS2 protein indicate that the amino but not the carboxy terminus of the protein is critical for RNA-protein interactions. J. Virol..

[91] Blaney J.E., Johnson D.H., Firestone C.Y., Hanson C.T., Murphy B.R., Whitehead S.S. (2001). Chemical mutagenesis of dengue virus type 4 yields mutant viruses which are temperature sensitive in vero cells or human liver cells and attenuated in mice. J. Virol..

[92] Blaney J.E., Johnson D.H., Manipon G.G., Firestone C.Y., Hanson C.T., Murphy B.R., Whitehead S.S. (2002). Genetic basis of attenuation of dengue virus type 4 small plaque mutants with restricted replication in suckling mice and in SCID mice transplanted with human liver cells. Virology.

[93] van Gennip R.G., van de Water S.G., van Rijn P.A. (2014). Bluetongue virus nonstructural protein NS3/NS3a is not essential for virus replication. PLoS ONE.

[94] Zwart L., Potgieter C.A., Clift S.J., van Staden V. (2015). Characterising Non-Structural Protein NS4 of African Horse Sickness Virus. PLoS ONE.

[95] Wall G.V., Wright I.M., Barnardo C., Erasmus B.J., van Staden V., Potgieter A.C. (2021). African horse sickness virus NS4 protein is an important virulence factor and interferes with JAK-STAT signaling during viral infection. Virus Res..

[96] Li Z., Lu D., Yang H., Li Z., Zhu P., Xie J., Liao D., Zheng Y., Li H. (2021). Bluetongue virus non-structural protein 3 (NS3) and NS4 coordinatively antagonize type interferon signaling by targeting STAT1. Vet. Microbiol..

[97] Fablet A., Kundlacz C., Dupre J., Hirchaud E., Postic L., Sailleau C., Breard E., Zientara S., Vitour D., Caignard G. (2022). Comparative Virus-Host Protein Interactions of the Bluetongue Virus NS4 Virulence Factor. Viruses.

[98] Gribaudo G., Lembo D., Cavallo G., Landolfo S., Lengyel P. (1991). Interferon action: Binding of viral RNA to the 40-kilodalton 2′-5′-oligoadenylate synthetase in interferon-treated HeLa cells infected with encephalomyocarditis virus. J. Virol..

[99] Hassel B.A., Zhou A., Sotomayor C., Maran A., Silverman R.H. (1993). A dominant negative mutant of 2-5A-dependent RNase suppresses antiproliferative and antiviral effects of interferon. EMBO J..

[100] Chakrabarti A., Jha B.K., Silverman R.H. (2011). New insights into the role of RNase L in innate immunity. J. Interferon Cytokine Res..

[101] Malathi K., Saito T., Crochet N., Barton D.J., Gale M., Silverman R.H. (2010). RNase L releases a small RNA from HCV RNA that refolds into a potent PAMP. RNA.

[102] Gusho E., Baskar D., Banerjee S. (2020). New advances in our understanding of the “unique” RNase L in host pathogen interaction and immune signaling. Cytokine.

[103] Loo Y.M., Fornek J., Crochet N., Bajwa G., Perwitasari O., Martinez-Sobrido L., Akira S., Gill M.A., Garcia-Sastre A., Katze M.G. (2008). Distinct RIG-I and MDA5 signaling by RNA viruses in innate immunity. J. Virol..

[104] Bivalkar-Mehla S., Vakharia J., Mehla R., Abreha M., Kanwar J.R., Tikoo A., Chauhan A. (2011). Viral RNA silencing suppressors (RSS): Novel strategy of viruses to ablate the host RNA interference (RNAi) defense system. Virus Res..

[105] Beattie E., Kauffman E.B., Martinez H., Perkus M.E., Jacobs B.L., Paoletti E., Tartaglia J. (1996). Host-range restriction of vaccinia virus E3L-specific deletion mutants. Virus Genes..

[106] Chang H.W., Uribe L.H., Jacobs B.L. (1995). Rescue of vaccinia virus lacking the E3L gene by mutants of E3L. J. Virol..

[107] Mortola E., Noad R., Roy P. (2004). Bluetongue virus outer capsid proteins are sufficient to trigger apoptosis in mammalian cells. J. Virol..

[108] Ishigaki S., Fonseca S.G., Oslowski C.M., Jurczyk A., Shearstone J.R., Zhu L.J., Permutt M.A., Greiner D.L., Bortell R., Urano F. (2010). AATF mediates an antiapoptotic effect of the unfolded protein response through transcriptional regulation of AKT1. Cell Death Differ..

[109] Hopker K., Hagmann H., Khurshid S., Chen S., Hasskamp P., Seeger-Nukpezah T., Schilberg K., Heukamp L., Lamkemeyer T., Sos M.L. (2012). AATF/Che-1 acts as a phosphorylation-dependent molecular modulator to repress p53-driven apoptosis. EMBO J..

