# Supplementary material for: Orbivirus NS4 Proteins Play Multiple Roles to Dampen Cellular Responses
Source: Viruses. 2023 Sep 12;15(9):1908. doi: 10.3390/v15091908 (PMC10535134; doi:10.3390/v15091908)
Supplement: Supplementary file 1 [file viruses-15-01908-s001.zip › NS4 supplementary data revision 4.pdf]

Supplementary figures and tables for manuscript **"Orbivirus NS4 Proteins Play Multiple Roles to Dampen Cellular Responses"** Mohd Jaafar et al.

```
1                   21                  41                  61                  81
|                   |                   |                   |                   |
GTAATGCAAGAGATCAGCATGGCGCAGCATGTGATGATGGCACCAGGCGATCTTATTCTGGAAGTTGAGGACCAGATACGGAGGAGAGGT
  -  C  K  R  S  A  W  R  S  M  -  -  W  H  Q  A  I  L  F  W  K  L  R  T  R  Y  G  G  E  V

          101                 121                 141                 161
          |                   |                   |                   |
ATGGCGGTTCATGTGTTACAACAGGATGGCGAGGGAACCAAATCTCGCCGTTCTTCCGCCGACCTTACCTCTTCCATTCCATCTCATTCA
  W  R  F  M  C  Y  N  R  M  A  R  E  P  N  L  A  V  L  P  P  T  L  P  L  P  F  H  L  I  H

181                 201                 221                 241                 261
|                   |                   |                   |                   |
CCGGCCGTTTCCACCCCTGAGGTATCGGAATATCTGAATCGAGCGTTTAACCTTAATGTGGGGTCGTATGACGAGGGTCCATTGCCAGAA
  R  P  F  P  P  L  R  Y  R  N  I  -  I  E  R  L  T  L  M  W  G  R  M  T  R  V  H  C  Q  K
                                  TCA(S)
                                  TTA(L)
                                  AGA(R)
                                  CGA(R)
                                  GGA(G)
                                  TGC(C)
                                  TGT(C)
                                  TGG(W)
          281                 301                 321                 341
          |                   |                   |                   |
GGCCCTCCCATCTTCCTTTCGCTCAAAGGATCCATCCTCAATGAGGCCAACCTTCTTCCTAGTGCCATTCGAAAGATGGCCTCCGTTCGT
  A  L  P  S  S  F  R  S  K  D  P  S  S  M  R  P  T  F  F  L  V  P  F  E  R  W  P  P  F  V

361                 381                 401                 421                 441
|                   |                   |                   |                   |
TCTACCTATGAGAAGGCTTTGAAAAAGTCCAATCTCAAGGTTTTCGAGTATCCTCAAACTGTTCGGACGCAAGACGAGCTCCTCAATGAG
  L  P  M  R  R  L  -  K  S  P  I  S  R  F  S  S  I  L  K  L  F  G  R  K  T  S  S  S  M  R

          461                 481                 501                 521
          |                   |                   |                   |
ATAAAACACTCAGAGAGGGGTGCTTCTTCTACACGACGTAGGCAGAAACGGGTGGATCTAGTGACAAACGATCCGAGTTACGTCCAAGAG
  -  N  T  Q  R  G  V  L  L  L  H  D  V  G  R  N  G  W  I  -  -  Q  T  I  R  V  T  S  K  R

541                 561                 581                 601                 621
|                   |                   |                   |                   |
GCGGCGGCATACTACTCGGCTCCTACCACTGATCCTACTTGGAAAGCCACATTACGTCGTATTCATGCTCAAGGAACTATAGTGGGTGGA
  R  R  H  T  T  R  L  L  P  L  I  L  L  G  K  P  H  Y  V  V  F  M  L  K  E  L  -  W  V  E

          641                 661                 681                 701
          |                   |                   |                   |
GCCTATGATCAAGAAATTTCTCCCAAACCTCCCTTCGAAGCCTTCTTGGATTTCCTTGACTCCAGGGGATGAGCACTCCCTCTCGCACAC
  P  M  I  K  K  F  L  P  N  L  P  S  K  P  S  W  I  S  L  T  P  G  D  E  H  S  L  S  H  T

721                 741                 761
|                   |                   |
GCCCCCCGCGTGATAGACGGGTAGTCATGCATCTCTTCTCATAC
  P  P  A  -  -  T  G  S  H  A  S  L  L  I
```

Figure S1. Sequence of genome segment 9 of St Croix River virus showing the ORF of NS4 (highlighted in yellow) interrupted by a TGA stop codon (in red at position 215-217). The eight possible codons which restore the ORF are shown, encoding six possible amino acids.

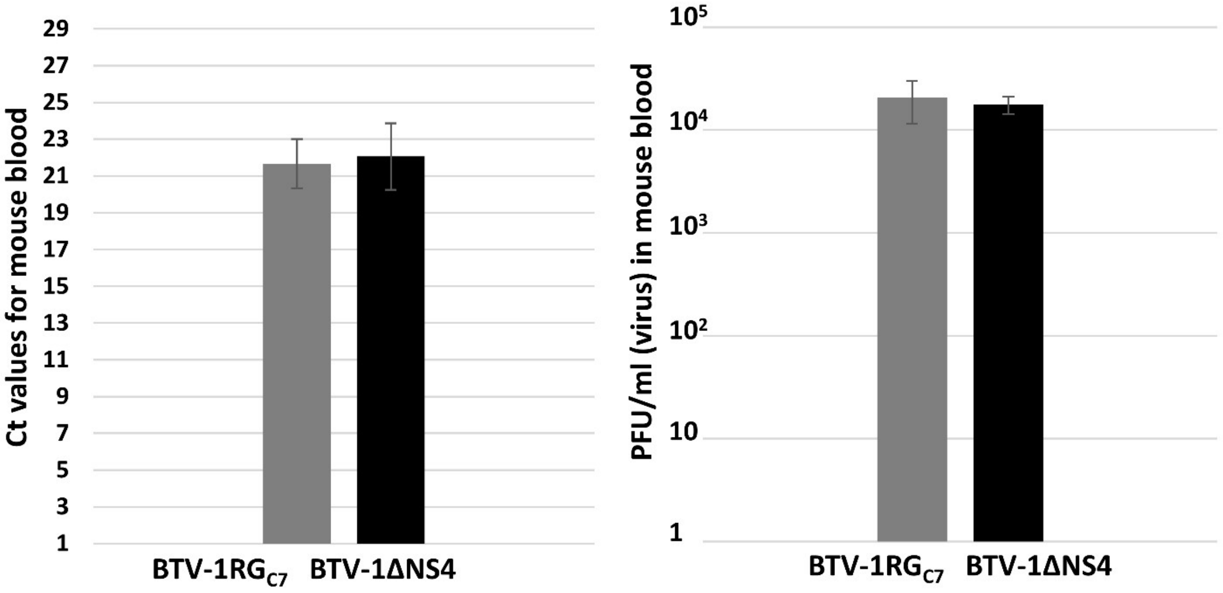

Figure S2. Ct values for viral RNAemia determined by RT-PCR and virus titres expressed in pfu/ml determined by plaque assay in mice inoculated with BTV-1RG$_{C7}$ or BTV-1ΔNS4.

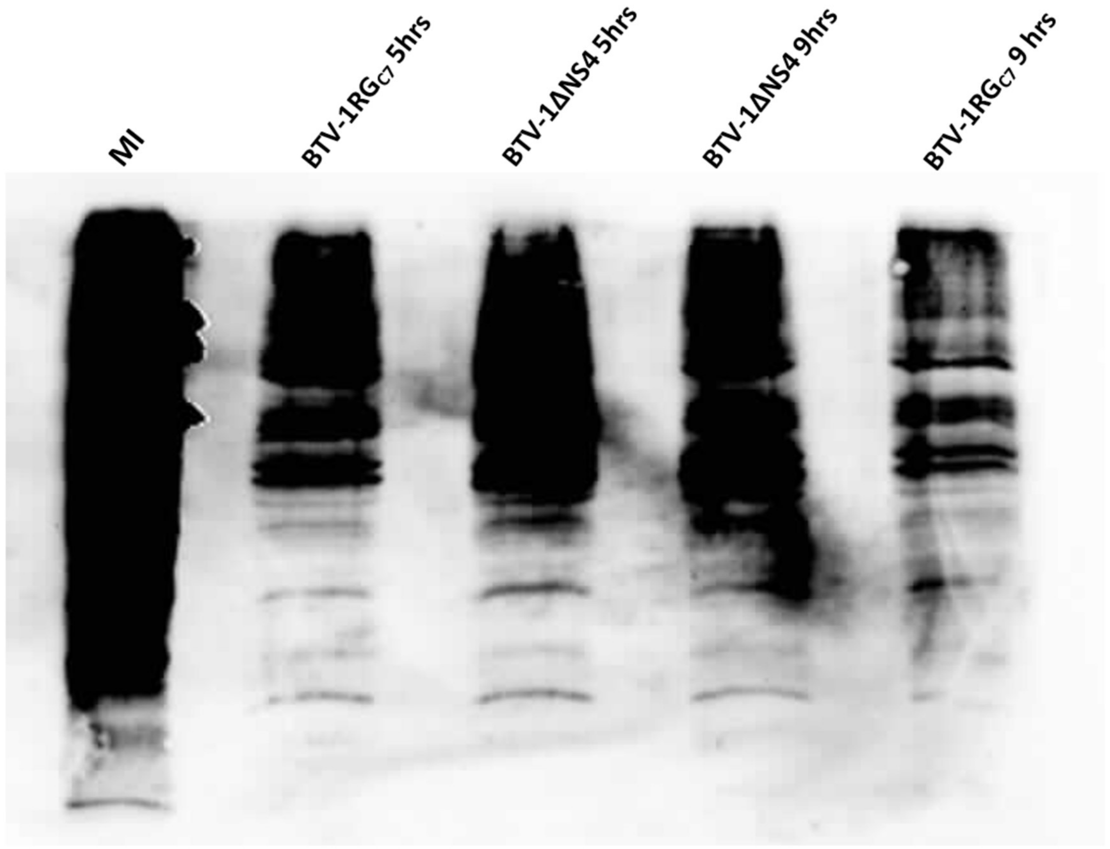

Figure S3. Pulse / chase metabolic labelling of BSR cells infected with BTV-1RG$_{C7}$ or BTV-1ΔNS4. This figure is identical to figure 3 in the main text, however with a longer exposure of the blot upon chemiluminescent detection. It depicts pulse / chase metabolic labelling of BSR cells infected with BTV-1RG$_{C7}$ or BTV-1ΔNS4 at 5 h and 9 h p.i. using L-azidohomoalanine (a methionine analogue) as label. MI: mock-infected.

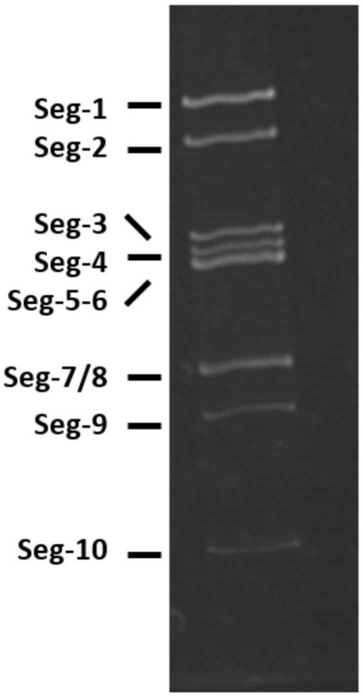

Figure S4. Purified dsRNA of Great Island virus, analysed by polyacrylamide gel electrophoresis using a 7.5% acrylamide gel.

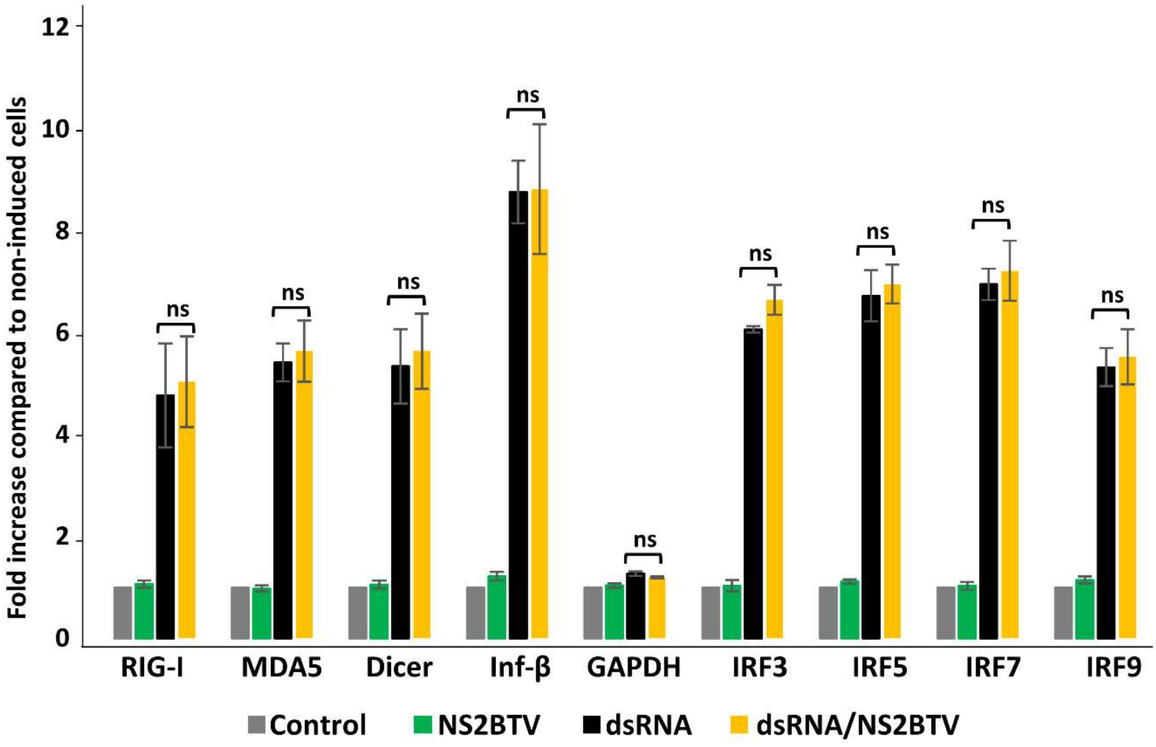

Figure S5. Comparison of expression of innate immune genes in HeLa cells induced by purified dsRNA from GIV-infected BSR cells (dsRNA) in the absence or presence of BTV-1 NS2 protein. GAPDH was included as a control gene (not involved in innate immunity). These experiments were conducted as three separate biological replicates. ns= not significant (p>0.5).

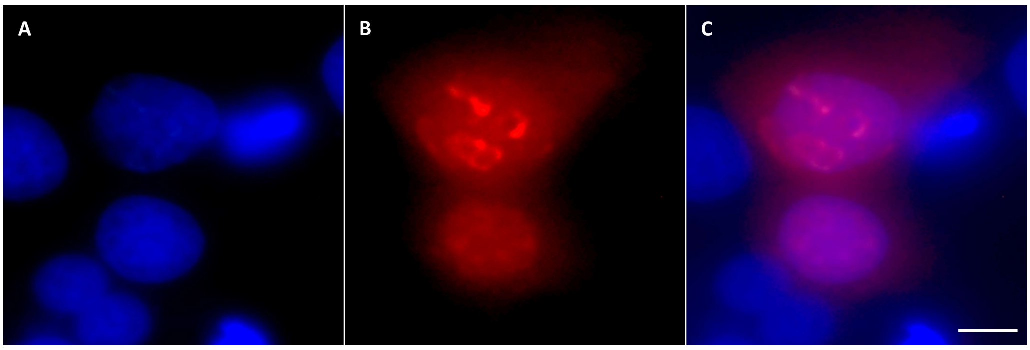

Figure S6. BSR cells transfected with plasmid pCIBTV1NS4 (at 24 h post-transfection). **A:** Nuclei stained with DAPI (blue), **B:** NS4 expression in the nucleus and cytoplasm detected by anti-NS4 antibodies and Alexa Fluor 568-conjugated IgG (red) and **C:** merged **A** and **B**. The scale bar represents 5 μm.

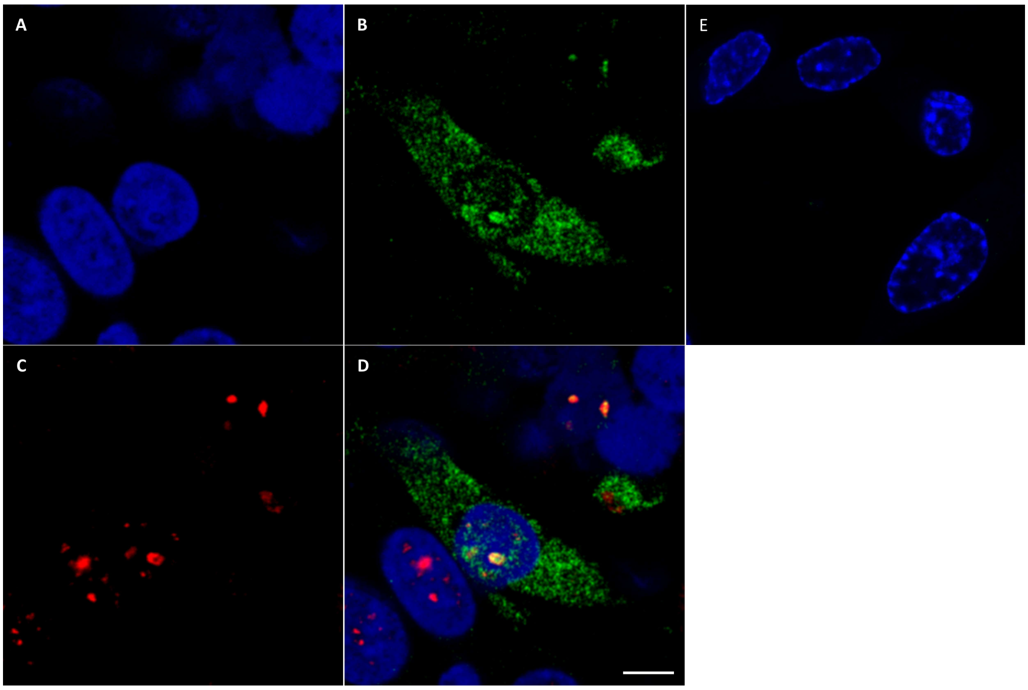

Figure S7. BSR cells transfected with plasmid pCIBTV1NS4 and assessed by FAM-FLICA at 24 h post-transfection. **A:** Nuclei stained with DAPI (blue), **B:** FAM-FLICA staining (green) of cells expressing NS4 indicating activation of caspases in transfected cells, **C:** NS4 expression detected by anti-NS4 antibodies and Alexa Fluor 568-conjugated IgG (red), **D:** merged **A, B** and **C** showing that NS4 localises with caspases in the nucleus (see Movie 1 for the z-stack, showing a wider field) and **E:** mock-transfected cells. The scale bar represents 5 μm.

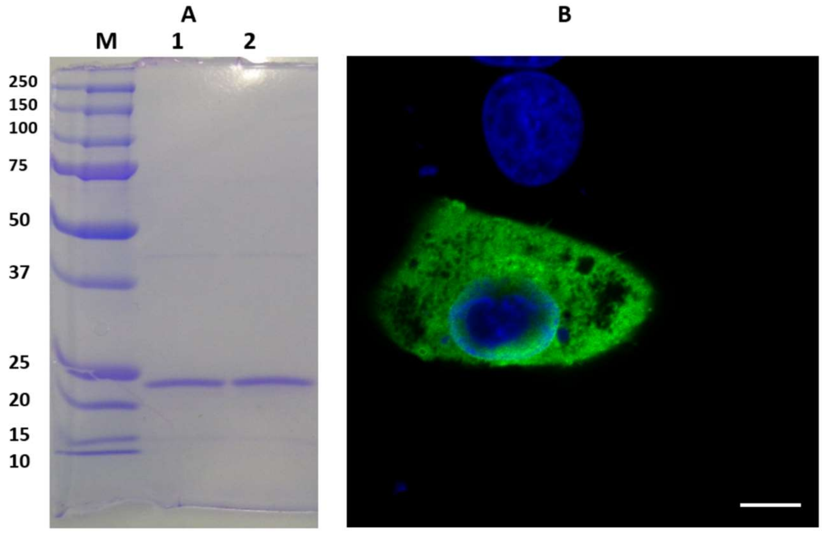

Figure S8. Expression of CIRV P19 in BSR cells transfected with plasmid pCIP19-6xHis. **A:** SDS-PAGE and Coomassie blue staining of pCIP19-6xHis protein purified using nickel-coated magnetic beads. **B:** confocal immunofluorescence using mouse anti-pentahis antibodies followed by anti-mouse Alexa Fluor 488-conjugated IgG. Nuclei are stained blue with DAPI.

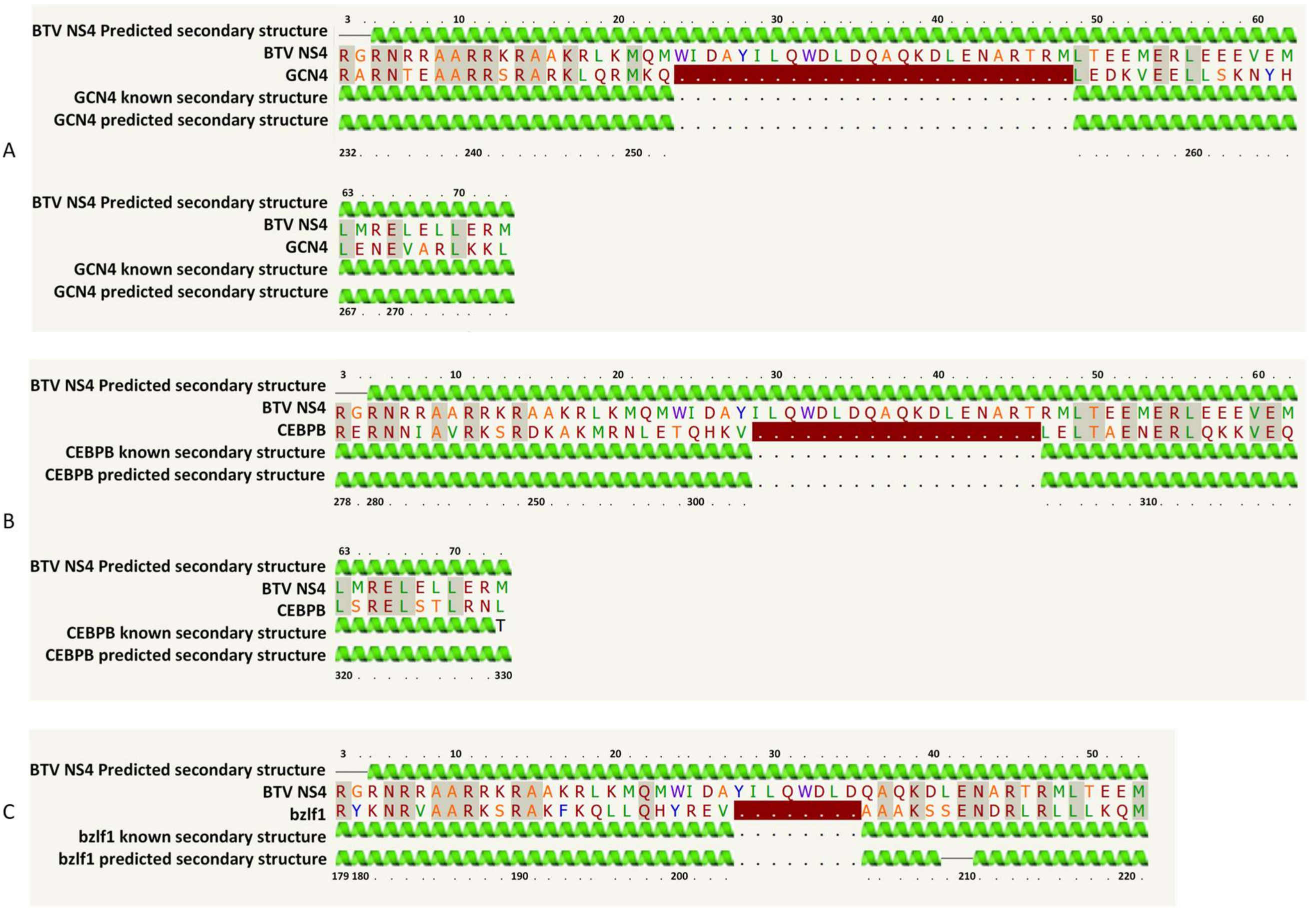

Figure S9. Secondary structure predictions for the amino acid sequence of BTV NS4 using Phyre2. The templates identified by Phyre2 are transcriptional regulators which include the general control of amino-acid synthesis like protein 4 or GCN4 (A), the transcription factor c/ebp beta or CEBPB (B) and the Epstein-Barr bzlf1 trans-activator protein (C).

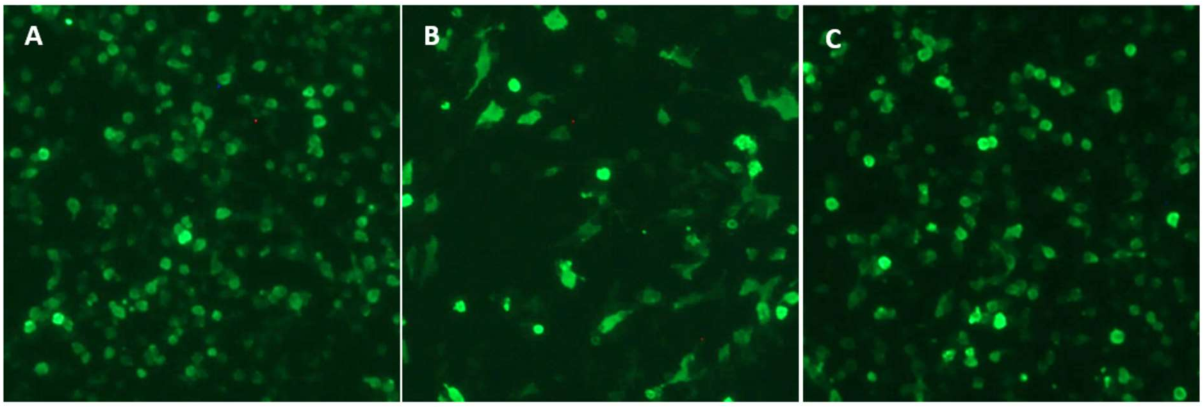

Figure S10. HEK293T cells transduced with TAT-tagged proteins. Cells were transduced with TAT-NS4BTV1-6xHis (**A**), TAT-HA-VP3BTV1-6xHis (**B**) or TAT-HA-NS4SCRV-6xHis (**C**) tested with anti-NS4 (**A**) or anti-HA tag antibodies (**B** and **C**). The results show that cells were efficiently transduced with the TAT-tagged proteins.

Table S1. Primer sequences used for cloning into mammalian and bacterial expression plasmids.

Primer	Sequence (5'→3')	RE	Plasmid	Target
NS4BTV1for	tacgGAATTCACCATGGTGAGGGGACGCAATCG	EcoRI	pCI-neo	Seg-9 BTV-1
NS4BTV1rev	tgagGCGGCCGCTCACTACCCATCTTCCTCCATTCGCTC	NotI	pCI-neo	Seg-9 BTV-1
NS4BTV1-6xHisrev	tgagGCGGCCGCTCACTA***GTGATGGTGATGGTGATG***CCCATCTTCCTCCATTCGCTC	NotI	pCI-neo/pGEXT-4T-2	Seg-9 BTV-1
NS4GIVfor	CTATCGGAATTCACCATGAGTTACCGGCAGGAGCA	EcoRI	pCI-neo	Seg-9 GIV
NS4GIVrev	tgatGCGGCCGCTCACTATTGCTGAACGCACCTTGTCC	NotI	pCI-neo	Seg-9 GIV
TAT-NS4BTV1for	tacgGAATTCCC*TACGGCCGCAAGAAACGCCGCCAGCGCCGCCGC*ATGGTGAGGGGACGCAATCG	EcoRI	pGEXT-4T-2	Seg-9 BTV-1
TAT-HA-VP3for	tacgGAATTCCC*TACGGCCGCAAGAAACGCCGCCAGCGCCGCCGC*TATCCGTATGATGTTCCGGAT TATGCAATGGCTGCTCAGAATGAGCAACG	EcoRI	pGEXT-4T-2	Seg-3 BTV-1
VP3-6xHisrev	tgagGCGGCCGCTCACTA***GTGATGGTGATGGTGATG***CACAGTTGGCGCAGCCAGCTTGGTGC	NotI	pGEXT-4T-2	Seg-3 BTV-1
TAT-HA-NS4SCRV(R)for	tacgGAATTCCC*TACGGCCGCAAGAAACGCCGCCAGCGCCGCCGC*TATCCGTATGATGTTCCGGAT TATGCAATGTGTTACAACAGGATGGCGAG	EcoRI	pGEXT-4T-2	Seg-9 SCRV
NS4SCRV(R)-6xHisrev	tgagGCGGCCGCTCACTA***GTGATGGTGATGGTGATG***AAGCCTTCTCATAGGTAGAACGAAC	NotI	pGEXT-4T-2	Seg-9 SCRV
NS2BTV1For	tcagCCCGGGGTCATGGAGCAAAAGCAACGTAGA	XmaI	pCI-neo	Seg-8 BTV-1
NS2BTV1rev	tgagGCGGCCGCCTAAACGCCGACCGGCAATATGA	NotI	pCI-neo	Seg-8 BTV-1
P19-For	agctgGGATCCACCATGGAACGAGCTATACAAGGAAAC	BamHI	pCI-neo	P19 TBSV
P19-6xHisrev	tgagGCGGCCGCTCATTA***GTGATGGTGATGGTGATG***CTCGCTTTCTTTCTTGAAGGTTTC	NotI	pCI-neo	P19 TBSV
Sigma3MRV3for	tacgGAATTCGCAATGGAGGTGTGCTTGC	EcoRI	pCI-neo	Seg-S4 MRV3
Sigma3MRV3Rev	tgagGCGGCCGCTCATTAGCCAAGAATCATCGGATCGC	NotI	pCI-neo	Seg-S4 MRV3
IFNβ-PromKpnIfor	tacgGGTACCTTCTCAGGTCGTTTGCTTTCC	KpnI	pGL3	Human interferon promoter
IFNβ-PromXhoIrev	tacgCTCGAGGTTGACAACACGAACAGTGTC	XhoI	pGL3	Human interferon promoter

Underlined sequences are specific to the amplified sequence; Sequences in bold italics characters represent the 6xHis tag; Sequences in italics (non-bold) represent the TAT tag; Sequences in blue characters are restriction enzyme (RE) sites.; Sequences in lower case characters are non-specific nucleotides added for an efficient restriction enzyme digestion; Sequences in red in the reverse primers represent stop codons.

The GIV NS4 ORF (accession number HM543473) contains a naturally occurring NotI site 'GCGGCCGC', which we mutated to GCGACCGC to avoid truncation of the ORF during cloning; the mutation does not modify the amino acid sequence).

Table S2. Antibodies used in immunofluorescence and western blot analyses.

Primary antibody	Source	Dilution	Species in which antibodies were raised
Anti-NS4-BTV	Belhouchet et al., 2011	1/500	Rabbit
Anti-NS4-GIV	Belhouchet et al., 2011	1/500	Rabbit
Anti-Penta His	Qiagen, 34660	1/500	Mouse
Anti-Caspase 3	Santa-Cruz sc-7272	1/100	Mouse
**Secondary antibody**	**Source**	**Dilution**	**Species in which antibodies were raised**
Alexa Fluor 568 (red) goat anti-mouse	Thermo Fisher A-11031	1/250	Goat
Alexa Fluor 488 (green) goat anti-mouse	Thermo Fisher A-11001	1/500	Goat
Alexa Fluor 488 (green) donkey anti-rabbit	Thermo Fisher A-21206	1/500	Donkey
Alexa Fluor 568 (red)) goat anti-rabbit	Thermo Fisher A-11036	1/250	Goat
Anti-mouse peroxidase	Beckman IM0817	1/750	Goat
Anti-rabbit peroxidase	Sigma, A0545	1/500	Goat

Table S3. Primer sequences used for real time PCRs.

Primer	Sequence (5'→3')	RE	Target
CulicoDcr-2For	CATCTCCTTGCAACTGAAGACG	NA	*Culicoides* Dcr-2
CulicoDcr-2Rev	CGTCGAATCAGCTGTTTGGG	NA	*Culicoides* Dcr-2
Act1CulicoFor	GTTGCACCAGAAGAACATCCAG	NA	*Culicoides* Actin-1
Act1CulicoRev	CCAGTGGTACGACCTGAAGC	NA	*Culicoides* Actin-1
EMCVBS2	CGGCACAACCCCAGTGCCAC	NA	EMCV
EMCVBR2	CCAGATCAGATCCCATACAATG	NA	EMCV
CoxIHamFor	GATTTGGAAACTGACTTGTAC	NA	Hamster CoxI
CoxIHamrev	AGACTGTTCAACCAGTTCCAGC	NA	Hamster CoxI
NS4BTfor	GATCTGGATCAAGCGCAAAA	NA	NS4 BTV-1
NS4BTrev	ACCTTTCCATCTCCTCTGTCAACA	NA	NS4 BTV-1
NS4BTProb	[FAM]ACCTGGAGAACGCGCGAACGAGA[TAMRA]	NA	NS4 BTV-1
NS4GIVfor	ACGAGTCCTCGGGTCTGAAAT	NA	NS4 GIV
NS4GIVrev	TGACCAACTCCGAGCTCCTT	NA	NS4 GIV
NS4GIVProb	[FAM]CCTATTCCGGATAGAGATCGCGTCCTGTT[TAMRA]	NA	NS4 GIV
VACV_forward	CCGTCCAGTCTGAACATCAATC	NA	Vaccinia virus
VACV_reverse	ACAAATAGAAAAGTGTTGTAAACGCAA	NA	Vaccinia virus
VACV_Probe	[FAM]CCAACCTAAATAGAACTTCAT[TAM]	NA	Vaccinia virus
SCRVFor1	CGGGTCGCCACGCTTAT	NA	SCRV
SCRVRev1	ACAGCGGAACGCTCAGAGAA	NA	SCRV
SCRVProbe1	[FAM]CCTCCCACCGTTCCCGCACTG[TAMRA]	NA	SCRV

Table S4. Ct values and virus titres in blood of mice infected with BTV-1RG$_{C7}$ or BTV-1ΔNS4 at day 4 post-infection (p.i.).

Virus	Ct value Day 4 p.i.	Viraemia (plaque assay)
BTV-1RG$_{C7}$	19.6-23.3 (mean = 21.66)	$1.2x10^4$-$3.5x10^4$ pfu/ml (mean=$2.1x10^4$)
BTV-1ΔNS4	19.1-23.8 (mean=22.06)	$1.4x10^4$-$2.3x10^4$ pfu/ml (mean=$1.76x10^4$)
